# MOV&RSim: computational modelling of cancer-specific variants and sequencing reads characteristics for realistic tumoral sample simulation

**DOI:** 10.1186/s12859-025-06292-0

**Published:** 2025-11-27

**Authors:** Francesca Longhin, Giacomo Baruzzo, Enidia Hazizaj, Diego Boscarino, Dino Paladin, Barbara Di Camillo

**Affiliations:** 1 Department of Information Engineering, University of Padova, Via Gradenigo 6b, 35131 Padua, Veneto Italy; 2 AB ANALITICA, Srl, Via Svizzera 16, 35127 Padua, Veneto Italy; 3 Department of Comparative Biomedicine and Food Science, University of Padova, Viale dell’Università 16, 35020 Legnaro, Veneto Italy; 4 Padova Center of Network Medicine, Via Marzolo 8, 35131 Padua, Veneto Italy

**Keywords:** Realistic simulation, Cancer-specific presets, Genetic variants, Tumoral clonality, Sequencing reads, Tumoral heterogeneity, Variants characteristics, Reads characteristics, Somatic sample simulator, Gold-standard

## Abstract

**Background:**

Bioinformatics pipelines for variant calling have undergone significant advancements due to the decreasing costs of next-generation sequencing. Accurate mutation detection is crucial for personalised medicine in cancer, particularly in assignment of therapy. Somatic variant calling, however, remains challenging due to diverse cancer types, heterogeneity, complex mutational profiles, and unpredictable sequencing errors. A dataset of fully characterised tumoral genomes and sequencing reads, large enough to represent the variability inherent in different cancer types, is still lacking, even considering synthetic data. The lack of such datasets hampers rigorous evaluation, benchmarking and optimization of variant callers for specific cancer types.

**Results:**

The contribution of this work is twofold. First, we conducted a comprehensive analysis of nine somatic sample simulators (Synggen, BAMSurgeon, SVEngine, VarSim, Xome-Blender, tHapMix, Pysim-sv, SCNVSim, HeteroGenesis) assessing their ability to control *biological parameters*, including variants characteristics (type, number, position, length, content, zygosity), and sample characteristics (clonality, contamination); and *technical parameters*, including reads characteristics (sequencing errors, coverage, base qualities). No single simulator provided complete control over both biological and technical parameters, nor guidance on tuning biological parameters for cancer-specific simulations. Consequently, we developed MOV&RSim, a novel simulator that leverages data-driven information to set variants and reads characteristics, producing realistic tumoral samples, and providing full control on biological and technical parameters. Additionally, we leveraged well-annotated variant databases to create cancer-specific presets that inform the simulator’s parameters for 21 cancer types.

**Conclusion:**

This new simulator, containerised with Docker and freely available for academic use, empowers users to define each biological parameter of a tumoral genome and faithfully replicates the variability of technical noise observed in real sequencing reads. The proposed simulator and presets represent the most adaptable and comprehensive framework currently available for generating tumor samples, enabling comprehensive benchmarking and, ultimately, the optimization of somatic variant callers across diverse cancer types.

## Background

Many disorders are caused by genetic variants [1–3]. Therefore, in clinical settings, it is essential to accurately identify mutational patterns within the human genome. In the past few years, remarkable progress has been made in developing and optimising bioinformatics pipelines for germline variant calling. The rapidly decreasing cost of next generation sequencing (NGS) technologies and the availability of gold-standard sample datasets, for example from Illumina Platinum Genomes [4] and Genome in a Bottle consortium [5], have enabled the creation of cutting-edge tools for detecting a wide range of genomic aberrations [6–8] and the evaluation of their performance at a large scale [9, 10]. Despite the current technical limitations associated with the main sequencing platforms, including sequencing errors [11] and uneven coverage [12], germline variant calling has reached a performance level that firmly consolidates its use for clinical decision-making when addressing inherited disorders [13]. Conversely, somatic variant calling introduces additional complexities with respect to germline variant calling. Indeed, cancer genomes exhibit significant heterogeneity, which can be categorised into four distinct types [14, 15]:

- *Inter-tumour heterogeneity* due to the great variety of cancer types and subtypes. The number [16] and types [12] of somatic variants highly depend on the cancer type considered.
- *Intra-tumour heterogeneity* due to clonality. Tumours can be composed of a heterogeneous mix of cells with different genetic mutations and characteristics. Clonal variants show inconsistent allele frequencies [17], which often leads to their misinterpretation as sequencing artifacts.
- *Inter-patient heterogeneity* due to the distinctive germline mutational profiles unique to each individual patient. These profiles play a role not only in determining cancer risk, but also in influencing tumour progression [18, 19].
- *Intra-patient heterogeneity* between primary and metastatic sites. Many evolutionary mutations help tumor cells adapt to changes in the micro-environment, and successfully colonising [15, 20].

Currently, there is the lack of a comprehensive dataset of gold-standard tumoral samples to be used for the purpose of benchmarking and optimising somatic variant calling methods for each tumour scenario [21]. Two gold-standard tumor sample datasets are available, COLO829 [22] and HCC1395 [23], but they represent only melanoma and breast cancer, respectively. Consequently, they do not provide a sufficiently exhaustive representation of cancer heterogeneity.

Nevertheless, somatic variant detection already proved to be essential in the study of certain tumor types [24, 25]. Therefore, to evaluate the performance of somatic variant calling pipelines in different tumoral scenarios, a comprehensive dataset of gold-standard samples, considering both the biological variability of tumoral genomes and technical limitations of NGS sequencing is needed.

There are several methods which are typically employed to obtain gold-standard samples (confirmation of variant calls through Digital Polymerase Chain Reaction [26], Sanger sequencing experiments [27], concordance of multiple variant callers [28], generation of synthetic data [29]). However, the only cost-effective approach to produce a large number of gold-standard samples, while also controlling their biological and technical characteristics is in-silico simulation. While in-silico sequencing allows fine control over technical parameters such as read length and coverage, simulation tools that are not properly configured may fail to reflect the complexity of real sequencing errors and the highly specific mutational profiles of tumoral genomes [21].

### Review of state-of-the-art somatic sample simulators

**Fig. 1:**
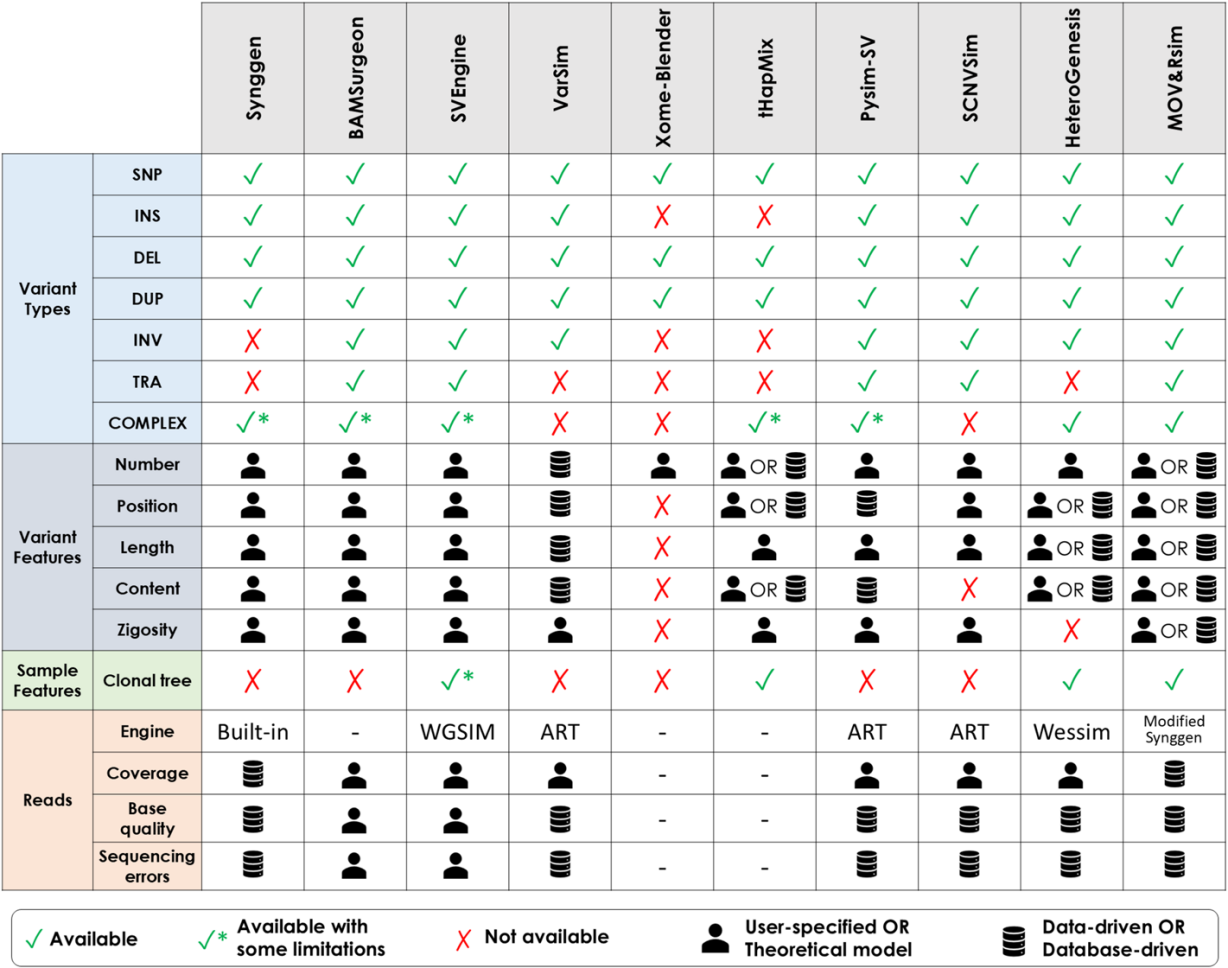
Overview of the main simulation features of 9 somatic sample simulators and MOV&RSim. For each simulator, the figure summarizes the types of variants it can simulate and the level of control it offers over variant-specific features. The ability to input a clonal phylogenetic tree is also indicated. In addition, the figure describes how sequencing reads are generated, including key technical parameters and how they can be configured. Further details regarding the degree of user control and the available configuration options for these simulation features are provided in Appendix A, Table A1 and Table A2

To better understand pros and cons of current simulation approaches, we assessed nine existing tumoral sample simulators (Synggen [30], BAMSurgeon [31], SVEngine [32], VarSim [33], Xome-Blender [34], tHapMix [35], Pysim-sv [36], SCNVSim [37], and HeteroGenesis [38]) for their completeness in designing cancerous genomes and the corresponding NGS reads. We compared them in terms of simulation approach used to introduce variants, degree of user control over available parameters, considering both parameters that model the complexity of the tumoral genome (*biological parameters*), as well as the noise caused by library preparation and sequencing (*technical parameters*), and setting modalities. Additionally, we reviewed the different types of variants that can be introduced with each simulator (Fig. 1): Single Nucleotide Polymorphism/s (SNP), Insertion/s (INS), Deletion/s (DEL), Duplication/s (DUP), Inversion/s (INV), Translocation/s (TRA), and complex overlapping variants. For DELs and DUPs, we attributed simulation capability to a given tool only if it could simulate both short (< 50 nucleotides) and long events (>50 nucleotides), which results in Copy Number Variant/s (CNV) [39].

A detailed comparison analysis is presented in Appendix A, Table A1, Table A2, and it is summarized in Fig. 1. The analysis showed that there is no individual simulator that provides simultaneously:

- High-level user control over all variant characteristics for all variant types (characteristic provided by BAMSurgeon and SVEngine);
- The possibility to set variant characteristics using data-driven information (characteristic provided by tHapMix);
- The possibility to incorporate variants in sequential order to manage complex overlapping events (characteristic provided by HeteroGenesis);
- A tree-oriented framework to define clones and their variants (characteristic provided by HeteroGenesis);
- The ability to mimic all read characteristics through multi-parametric empirically-derived models (characteristic provided by Synggen).

Overall, none of the available simulators individually provides control over all biological and technical characteristics of a tumoral sample, and it is often not clear how to fine-tune parameters to simulate specific cancer types. Ultimately, these limitations hinder the reliable and comprehensive evaluation of somatic variant callers using simulated data.

### Proposed solution

To address the limitations of current simulation approaches, we developed MOV&RSim, a novel simulator that extends the functionalities of state-of-art simulators by integrating some of their specific functionalities along with novel MOV&RSim-specific features. Furthermore, we derived data-driven information about somatic variants number, type, zygosity, lengths, and positions in cancer-specific samples from Catalogue Of Somatic Mutations In Cancer (COSMIC) [40] and The Cancer Genome Atlas (TCGA) [41]. We then stored such data in structured presets which can inform the proposed simulator, resulting in biologically realistic simulated genomes for each tumoral scenario.

The core strength and innovation of MOV&RSim reside in its ability to customise both biological and technical aspects of a simulated tumour sample, providing a way to set them realistically by learning biological information from COSMIC and TCGA, and technical information from real sequencing data. For this reason, MOV&RSim represents a valuable resource for comprehensively evaluating somatic variant callers.

The paper is organised as follows. Section “Results” introduces MOV&RSim, details the data-driven presets, and includes a case study using MOV&RSim. Sections “Discussion” and “Conclusions” draws some conclusions and outlooks for future work. Section “Implementation” details the algorithm, the implementation and the settings of MOV&RSim.

## Results

### MOV&RSim: towards a more complete simulator of tumoral samples

**Fig. 2:**
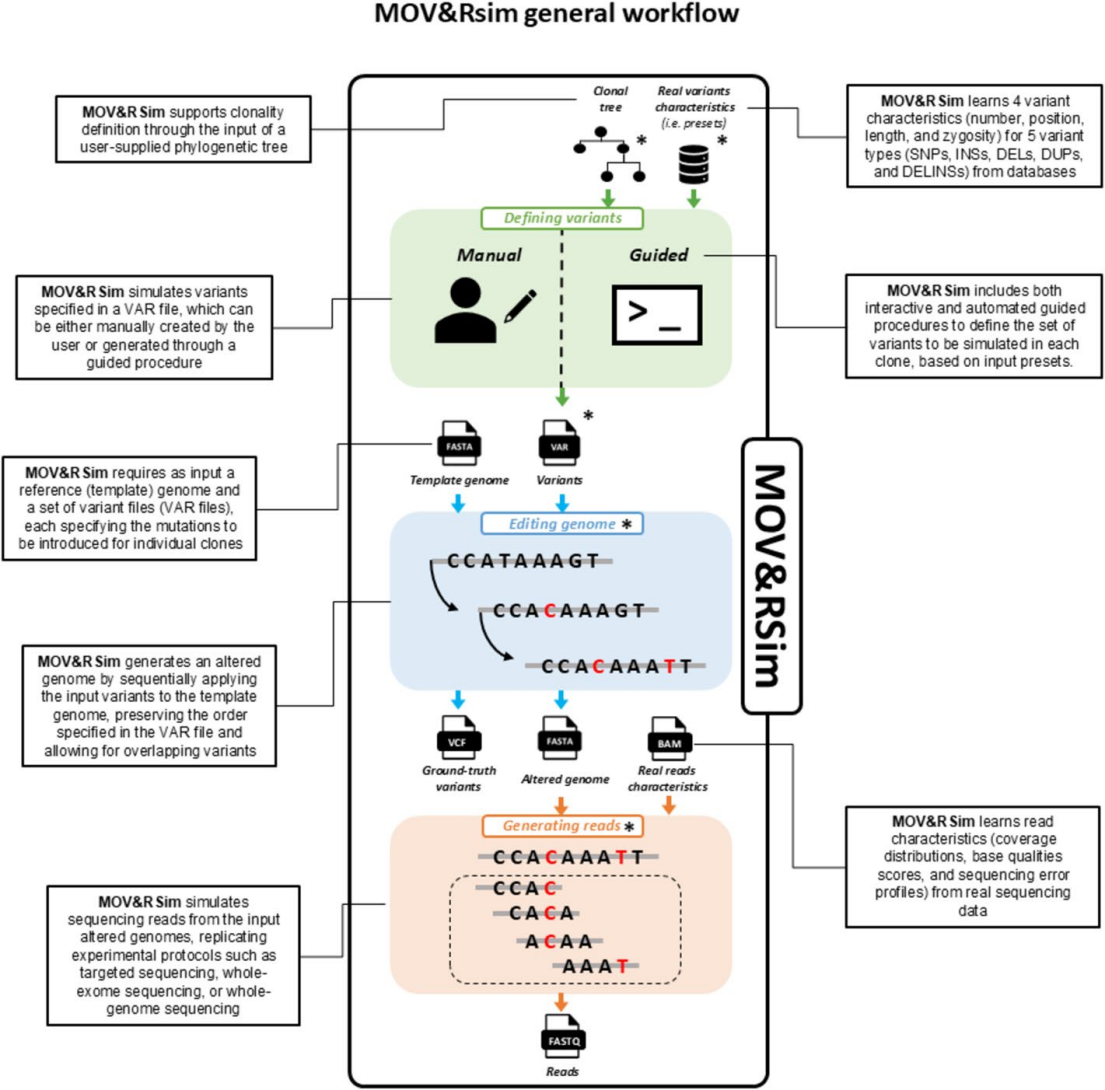
Graphical representation of MOV&RSim simulation workflow. MOV&RSim enhances formats and functionalities from five existing simulators (denoted with *). In the first simulation phase (green box), the user defines variants in the VAR format, a file format inspired by BAMSurgeon and SVEngine. VAR files can be compiled manually or using a built-in guided procedure, which optionally takes as input a clonal tree architecture in the HeteroGenesis format and information about real variants characteristics, similar to tHapMix. In the second simulation phase (blue box), MOV&RSim integrates variants into a template genome in sequential order, following the HeteroGenesis idea. In the third simulation step (orange box), using an adapted version of Synggen, MOV&RSim learns information about real reads characteristics and uses it to generate new reads

Here we present MOV&RSim, an innovative simulator that enhances the most valuable functionalities of existing simulators, standing out as the only tool that provides full user control over all *biological* and *technical parameters* (see Table A1, Table A2, and Fig. 2).

To ensure high-level user control over all variant types and variant characteristics, MOV&RSim uses an input file format similar to the VAR format, used in BAMSurgeon and SVEngine. It contains a list of variants that the simulator incorporates into a template genome to construct the mutated genome of the final simulated sample. Through the VAR file, MOV&RSim inherits BAMSurgeon and SVEngine’s ability to provide individual variant control over all variant characteristics (number, position, length, content, zygosity) for all variant types (SNPs, INSs, DELs, DUPs, INVs, TRAs, Complex) (see Sect. “Implementation” for more details). Yet, BAMSurgeon and SVEngine VAR formats have some constraints: when detailing the nucleotide content of insertion events with the BAMSurgeon VAR format, the user has to define the insertion sequence manually, while with the SVEngine VAR format the user can only draw it from an external genome file (e.g., to mimic viral DNA integration). Instead, MOV&RSim’s implementation of the VAR format not only accommodates both these approaches, but also allows the generation of random content, enhancing user convenience. Moreover, as the VAR file describes variants for each independent sample, MOV&RSim can accept multiple VAR files and parallelise the process of introducing their variants into copies of the same template genome to obtain many altered genomes simultaneously, one for each simulated sample, in a time and computationally efficient way. Additionally, MOV&RSim is equipped with a user-friendly guided procedure designed to simplify and accelerate the process of populating VAR files. This guided approach to generate the VAR file is an exclusive feature of MOV&RSim, contributing to error prevention (e.g., by displaying chromosome limits).

To give the possibility to set variant characteristics using data-driven information, MOV&RSim’s guided procedure for creating VAR files includes the option to fill each sample/clone’s VAR file using information about real variants characteristics, rather than relying solely on manual input. While tHapMix previously offered a similar feature for DELs and DUPs to set their length and zygosity, MOV&RSim expands this functionality to include additional variant types (SNPs, INSs, and combined Deletion/s–Insertion/s (DELINS)) and characteristics (number and position). Moreover, tHapMix computes distributions of variant characteristics from a list of variants, without considering information about the sample type to which each variant belong. Instead, MOV&RSim integrates presets derived from COSMIC [40] and TCGA [41], to accurately set variant characteristics in sample-specific simulations for 21 cancer types.

To provide the possibility to incorporate variants in sequential order and simulate complex overlapping events, MOV&RSim introduces mutations into the template sequence in the order specified in the input VAR file, building on the idea of HeteroGenesis. HeteroGenesis does not fully exploit the power of this feature as users can only control the rate of variants per type, with variants randomly selected from a pre-compiled list, limiting the control over the characteristics of each specific variant that is actually inserted. MOV&RSim, by providing full control over each individual variant, allows the nucleotide content of insertion events to be sampled directly from the sequence undergoing mutation. In this way, the insertion potentially carries previously introduced variants with known types and characteristics, achieving an unprecedented level of flexibility and customisation when designing overlapping events.

To enable the simulation of clones with a predefined tree-structure, MOV&RSim manages multiple VAR files simultaneously, one for each clone, generating them based on the input clonal tree architecture. The procedure takes a tree architecture specified using the HeteroGenesis format, as it allows users to indicate the number of variants that differentiate each parent–child pair in the phylogeny. Each child’s VAR file is composed of its parent’s VAR file plus the tree-defined number of newly generated variants unique to that clone, all with user-defined types and characteristics.

Last, to simulate realistic reads resembling empirically-derived NGS data characteristics, MOV&RSim relies on Synggen read simulation. Synggen is superior to other read simulators as it is the only one that models technical information (coverage distributions, base qualities, sequencing errors) from real, platform-specific reads, mapped to a reference genome. MOV&RSim leverages Synggen to generate synthetic reads in user-specified regions of interest, emulating Whole Genome Sequencing (WGS), Whole Exome Sequencing (WES), or Targeted Sequencing (TS) experiments. However, as real reads are mapped to a reference genome, in the original version of Synggen such information can only be used to generate synthetic reads from the same reference sequence. MOV&RSim implements an adapted version of Synggen that converts technical information to fit into mutated genomes, increasing Synggen’s flexibility.

#### MOV&RSim general workflow

The workflow of MOV&RSim is shown in Fig. 2 and described in details in Sect. “Implementation”. Overall, MOV&RSim can be summarized in 3 main blocks: (i) defining variants, (ii) editing genome, and (iii) generating reads.

The Defining Variants block helps the user to create an input VAR file, using MOV&RSim guided procedure and leveraging on real variant characteristics learn from COSMIC and TGCA databases, and stored in MOV&RSim preset. This block is optional and provided solely for user convenience. Users who already have their own VAR files, or who prefer to create them manually, can skip this step.

The Editing Genome block takes as input a reference genome and alters it based on the variants described in the input VAR file. For each sample/clone, MOV&RSim takes as input the given VAR file and generates the corresponding altered genome by independently modifying the two haplotypes: heterozygous variants are applied exclusively to the first haplotype, while homozygous variants are applied to both. This step provides as output all spiked-in variants in a ground-truth VCF file (for use in subsequent variant calling experiments), and the altered genome.

The Generating reads block uses the enhanced version of Synggen to simulate reads in FASTQ format for each haplotype of each sample/clone, starting from the altered genome produced in the previous step. Realistic read characteristics (i.e. coverage distributions, base qualities, sequencing errors) are learn from user-specified BAM files. The user can choose to simulate strand bias, purity, and clonality either by manually or automatically mixing the generated reads from each haplotype of each sample/clone. In the latter approach, MOV&RSim calculates the number of reads to be drawn from each haplotype of each sample/clone by asking the user for the total coverage, the proportion of reads from each haplotype (e.g., 0.5 and 0.5 for no strand bias), and the percentage of reads from each sample/clone (e.g., for 4 clones, values like 0.3, 0.2, 0.1, 0.4).

### A guided procedure for defining variants using data-driven presets

#### Data-driven presets from COSMIC and TCGA

In order to obtain reliable information about the characteristics of real variants in cancer-specific samples, and to allow users to use this information when defining variants for simulating a new sample, we calculated presets from COSMIC and TCGA data for 21 cancer types (see Sect. “Implementation” for the complete list of cancer types). COSMIC provides data on validated somatic variants identified through WGS, whereas TCGA focuses on somatic variants detected using WES protocols. The COSMIC database includes a larger number of samples compared to TCGA (see Table B3), offering greater statistical power for describing simulated datasets. Together, these databases represent data from the two most widely used workflows in NGS, providing users with presets that closely resemble the majority of currently available sequencing data.

Table B4 shows for each database and cancer type the proportions of each variant type, with variants classified into SNPs, INSs, DELs, DUPs, and DELINS. Table B5 displays the proportions of heterozygous and homozygous variants. Table B4 and B5 show that, for the same cancer type, the proportions of variants are relatively consistent between COSMIC and TGCA, while different cancer types show distinct mutational signatures in terms of type of variants and zygosity.

Not only are the types of variants highly specific to each cancer type, but the characteristics of individual variant types, such as their number, length, and position, also exhibit significant variability. Tables B6-B12 report the best fitting theoretical distributions for representing the total number of variants, variant lengths, and the positions per variant type for each cancer type and for each database. The best fitting theoretical distributions were evaluated with Akaike Information Criterion (AIC), Bayesian Information Criterion (BIC), Kolmogorov–Smirnov (KS), Anderson–Darling (AD), Cramer–von Mises (CvM), Chi Square (CHISQ) statistics, reported in tables B6-B11, respectively; table B12 reports the best fitting theoretical distributions based on majority voting across the different statistics. Different variant types and characteristics exhibit substantial variation in their best-fitting theoretical distributions across cancer types, highlighting a level of heterogeneity that must be accounted for in realistic simulation models.

Overall, tables B4-B11 highlight the importance of accounting for diverse variant characteristics when simulating different types of cancer. They underscore the necessity for users to access cancer-specific presets that accurately reflect these differences.

#### MOV&RSim’s guided procedure for defining variants

**Fig. 3:**
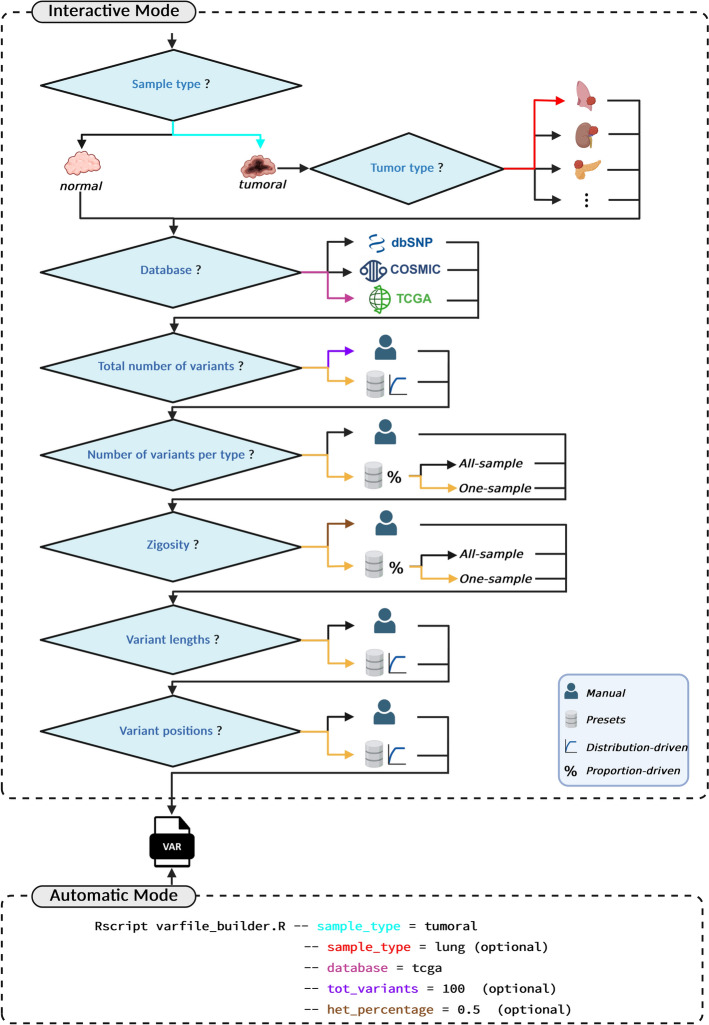
Overview of MOV&RSim’s guided procedure for defining variants and obtaining the corresponding VAR file. When running the interactive mode, the procedure begins by asking the user to select the tumour type to simulate and the database to rely on. The user is then given the option to manually set the total number of variants, the number of variants per type, variants zygosity, lengths, and positions, or rely on the presets. When using presets to define variant characteristics, two approaches can be applied depending on the specific characteristic being considered: the distribution-driven approach or the proportion-driven approach. For the proportion-driven approach, users can adopt either an all-sample perspective or a one-sample perspective. When running the automatic mode, the only mandatory flags are the tumour type (red arrow) to simulate and the database (pink arrow). By default (yellow arrows), the total number of variants, variants lengths, and positions are automatically selected, sampling the value/s from the empirical distributions, while the variants number per type and variants zygosity are selected using the proportions calculated with the one-sample perspective. Optionally, the user can specify the total number of variants (purple arrow) and/or the percentage of heterozygous variants (brown arrow)

The first step to characterise a tumoral sample is to define variants and their characteristics. However, state-of-art somatic samples simulators offer no guidance on how to set variant characteristics to obtain realistic simulations, specific to different cancer scenarios. Therefore, MOV&RSim provides a guided procedure (Fig. 3) for characterising variants using presets derived from COSMIC and TCGA data analysis. Supported variant types in the guided procedure are SNPs, INSs, DELs, DUPs, and DELINSs. Variant characteristics are the total number of variants, the number of variants per type, variants zygosity, variants lengths, and positions.

The procedure can run interactively or automatically. When running the interactive mode, users select the tumour type (21 available, see Table B3 for the complete list) and the preferred database (COSMIC, for resembling mutation data identified through WGS experiments, or TCGA, for WES). Then, users have the option to manually specify values for each variant characteristic or to rely on the presets. When relying on the presets, the setting modality depends on the specific variant characteristic being considered. When setting characteristics such as the total number of variants, variants lengths, and positions, value/s is/are drawn from distributions of those characteristics in the database (distribution-driven approach). Both the empirical distribution (total number of variants per sample, lengths of variants per variant type, positions of variants per variant type) and a set of theoretical distributions (Weibull, gamma, lognormal, etc. See Sect. “Implementation” for the complete list), fitted against the empirical data, are shown to the user in the form of Cumulative Density Functions (CDF) (Figure  B1).

The user chooses from which distribution (empirical or theoretical) to sample the value/s, based on visual inspection and on the suggestions of different goodness of fit metrics (AIC, BIC, KS, AD, CvM, CHISQ), displayed alongside the distributions. Conversely, when setting the number of variants per type and variants zygosity, characteristics that are constrained to the total number of variants, values are set using proportions (proportion-driven approach). Specifically, the user can choose whether to set the numerosity of SNPs, INSs, DELs, DUPs, DELINSs, and of heterozygous/homozygous variants to reflect the proportions calculated considering all the variants in the database (all-sample perspective) or those observed in a randomly selected sample (one-sample perspective). For example, suppose the user set 100 as the total number of variants, and that, in the chosen database, with the all-sample perspective, proportions are 50%, 10%, 20%, 10% and 10% for the 5 variants types, and 90% and 10% for the 2 zygosity types. The number of variants per type and of heterozygous/homozygous variants is determined accordingly, i.e. 50 SNPs, 10 INS, 20 DEL, 10 DUP, 10 DELINS, of which 90 in heterozygosity, 10 in homozygosity.

When running the automatic mode (Fig. 3), the user is only asked to specify on the command line the cancer type to be simulated and the database to be used. By default, the total number of variants, their lengths, and positions is/are selected using the distribution-driven approach, sampling the value/s from empirical distributions, while variant numbers per type and zygosity are selected using the proportion-driven approach with the single-sample perspective. Optionally, the user can specify the total number of variants and/or the percentage of heterozygous variants.

### Case study using MOV&RSim

To validate the proper functioning of the simulator, we created a toy VAR file (see Fig. C2) containing examples of the variants that can be simulated using MOV&RSim. These variants were incorporated into the template genome GRCh38, and paired-end reads were generated. As regions of interest for reads generation, we specified regions centered on the simulated variants (TS reads). To focus solely on assessing the correct incorporation of the variants, we considered a simplified scenario in which all variants are in homozygosity (VAF = 100%). Sequencing errors were disabled, and all regions of interest were assigned equal probabilities of being selected for the generation of a read. The simulated reads were then aligned using BWA-MEM2 [42] and visualised using the Integrative Genomics Viewer (IGV). Further details about data simulation are reported in Sect. “Case study settings”.

**Fig. 4:**
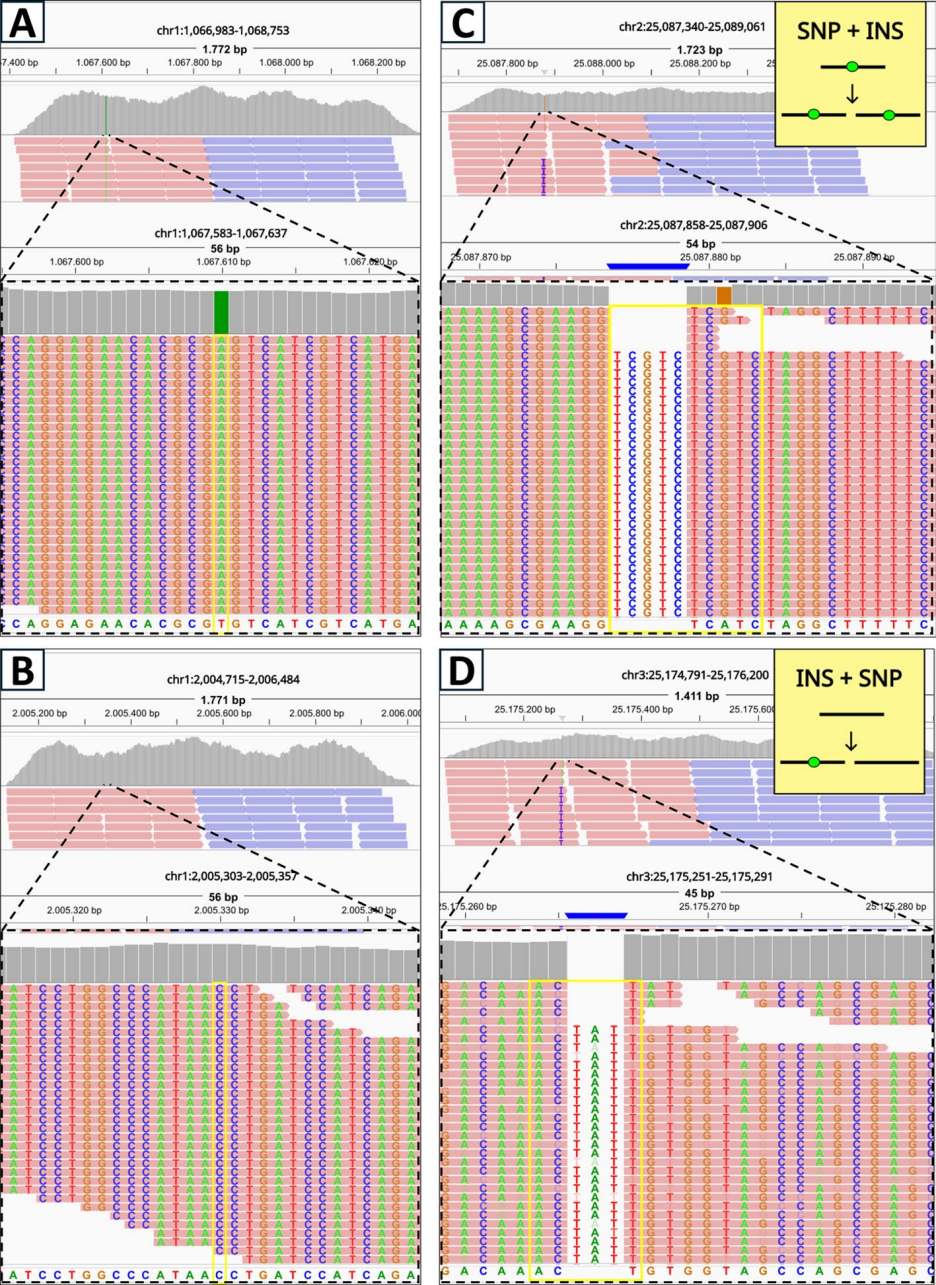
IGV visualisation of two simulated SNPs and two simulated complex variants. **A** A SNP defined by the user substitutes a "T" with an "A" at position 1’067’610 on chromosome 1. **B** A random SNP substitutes a "C" with a "C" at position 2’005’330 on chromosome 1. **C** A SNP defined by the user substitutes an "A" with a "G" at position 25’087’881 on chromosome 2. Subsequently, the user defines an INS in position 25’087’879 whose insertion sequence is the region altered by the SNP (nucleotides from 25’087’879 to 25’087’883). **D** The user defines an INS in position 25’175’263 of chromosome 3. The insertion sequence is the region from 25’175’264 to 25’175’266 ("ACT"). Subsequently, the user defines a SNP which substitutes the "C", which, before the INS, was in 25’175’265, with a "T"

As shown in Fig. 4 and in Fig. C3–C8, MOV&RSim correctly spikes-in the variants into the template genome and, consequently, in the generated reads.

Figure 4A, B depicts the simulation of two SNPs: in the first case (Fig. 4A), the substituting nucleotide is specified by the user ("A"), while in the second case (Fig. 4B), the nucleotide is selected randomly. By chance, this randomly chosen nucleotide is "C", which happens to match the nucleotide already present at that position in the template genome. This highlights a common issue faced by all simulators: as users typically do not check which nucleotides are present in the template genome when defining the nucleotide content of the variants they want to insert (either manually or through random selection), the ground truth may indicate the presence of a variant that is undetectable in practice. MOV&RSim is the only simulator that addresses this issue by flagging such SNP events, enabling easy filtering from the ground truth prior to downstream analysis.

Figure 4C, D depicts the simulation of two overlapping variants. In the first scenario (Fig. 4C), a SNP is inserted before an INS with the INS sequence derived from the region previously altered by the SNP. In the second scenario (Fig. 4D) an INS is simulated before a SNP, with the SNP subsequently modifying the INS sequence. This confirms that MOV&RSim takes into account the order in which variants are specified in the input file. Notably, the scenario shown in Fig. 4C, D cannot be reproduced by other simulators, as they lack the flexibility to model overlapping variants at this level of detail. For instance, SVEngine and BAMSurgeon can only generate compound variants starting at the same position, without modeling the temporal sequence of consecutive events. Tools such as tHapMix, Pysim-sv, HeteroGenesis, and Synggen consider overlapping variants over time but with important limitations: tHapMix does not support INS events; Pysim-sv offers limited user control over variant content and position; and HeteroGenesis and Synggen handle such cases only via CNVs (DEL and DUP $\ge$50 nucleotides), leaving their behavior uncertain for shorter events. In contrast, MOV&RSim provides a substantially more flexible and accurate framework for simulating overlapping variant scenarios.

Additional advanced examples of MOV&RSIM usage are described in Appendix D: *i)* simulation and analysis of cancer-specific mutational patterns using presets (Appendix D.1), *ii)* simulation of reads technical noise and its impact on variant calling performance (Appendix D.2), and *iii)* simulation of tumor clonality from input phylogenetic tree (Appendix D.3).

## Discussion

From a performance perspective, MOV&RSim offers clear advantages in both automation and efficiency compared to existing simulators. It is currently the only tool that provides a fully automated pipeline for input file generation ("Defining Variants"), significantly reducing manual workload and ensuring scalability for simulations involving large numbers of variants. MOV&RSim’s automated procedure for generating VAR files requires approximately 3 min and 5 GB of RAM for 1000 variants, and 5 min and 5 GB of RAM for 10,000 variants (Intel Xeon Gold 5118 2.30 GHz CPU).

The most computationally demanding step, “Editing Genome”, consumes around 50 GB of RAM per sample, taking $\sim$1 h for 1000 variants and $\sim$8 h for 10,000 variants. For comparison, Pysim-sv takes about 1 h on an Intel Xeon 2.40 GHz CPU and 10 GB of RAM to simulate only 100 variants [36], making it significantly less efficient than MOV&RSim. On the other hand, SVEngine and SCNVSim are reported to spike-in 15,000 variants in approximately 10 min and 2 h (Intel Xeon E7-4850 2.30 GHz CPU), respectively, faster than MOV&RSim, though their memory usage is not reported [32]. However, both simulators offer far fewer simulation features, configuration options, and user control than MOV&RSim (see Appendix A).

In the “Generating Reads” block, MOV&RSim invokes Synggen Mode 0, the converter, and Synggen Mode 1. When simulating 10x Illumina reads, Synggen Modes 0 and 1 complete in under 60 s, while the converter takes approximately 5 min. Although the state-of-the-art read simulator ART performs slightly faster, completing the task in about 300 s (Intel Xeon 2.93 GHz CPU) [43], this difference is minor. More importantly, MOV&RSim derives all read characteristics (coverage, base qualities, and sequencing errors) directly from real sequencing data, unlike ART, which models only base qualities and errors (see Appendix A and Tables  A1-A2). Moreover, ART relies on prebuilt empirical models, whereas MOV&RSim allows users to train models from their own reads, regardless of sequencing platform or library preparation. This provides a high level of flexibility, enabling simulations mimicking specific experimental conditions.

Despite the new simulation capabilities introduced by MOV&RSim, both the simulator and its presets have some limitations. As simulators’ users typically do not check which nucleotides are present in the reference genome when defining the nucleotide content of the variants they want to simulate, some simulated variants may comprise nucleotides that, by chance, mimic the reference genome. In such cases, it becomes impossible for variant callers to correctly identify these variants. This limitation affects all simulators. To address this issue, MOV&RSim includes a feature to automatically detect such cases (specifically for SNPs) which can be easily filtered out from the ground truth before downstream analysis.

COSMIC and TCGA presets contain statistical information about confirmed somatic variants. As such, when MOV&RSim defines new variants using presets information, it might produce easy to detect mutation data. On the other hand, learning presets from raw or unfiltered data could result in replicating errors rather than real variants. While presets are provided for user convenience, users have the flexibility to bypass them and manually configure variants if they find that the presets do not align with the specific needs of their experiments.

Since COSMIC and TCGA presets focus solely on somatic variants, they exclude germline variants. Similar to other simulators, creating a proper tumor sample involves creating a custom VAR file where the initial set of variants (germline) is manually defined, while the remaining somatic variants can be either manually defined or generated using the presets. This same approach applies when the user wants to simulate tumor-matched samples. Although the current presets are meant to simulate tumor-only data, the simulator can be executed twice (once using a manually created VAR file with germline variants, and once with a tumoral VAR file, either manually created or generated using presets) to simulate both the tumor and its matched normal control.

MOV&RSim allows to simultaneously control the mutational patterns of multiple clones which the user might wish to include into the same simulated sample, resulting in variants with extremely low VAFs. These events are often mistaken for sequencing errors in real case scenario, even when their correct detection would be essential to understand the mechanisms that control cancer progression, metastasis and resistance to chemotherapy. Therefore, it is essential to include these events in simulated genomes to effectively benchmark variant calling algorithms and select the ones that optimally detects them. While the case study presented in the main text focuses on simplified examples designed to illustrate the simulator’s functionality, a more realistic scenario involving heterogeneous clonal variants is provided in Appendix D (Section D.3), demonstrating the tool’s applicability to complex, real-world settings.

The added value of MOV&RSim lies in its broader scope and completeness, integrating functionalities that are fragmented or missing in existing simulators. We illustrated MOV&RSim’s capabilities through case studies (Section “Case study using MOV&RSim”, Appendix D). Remarkably, MOV&RSim is the only simulator able to reproduce the proposed scenarios, whereas other tools either lack the necessary simulation features or require ad hoc adaptations. Most prior simulator studies have offered primarily qualitative comparisons, typically by listing the strengths and limitations of each approach, since no established benchmarks or quantitative metrics currently exist to assess simulator quality. Our work also includes comparative analyses of simulation functionalities, but goes further by examining not only the presence or absence of specific features, but also their implementation and the degree of user control they allow (Appendix A). We argue that combining case studies with a systematic comparison of simulation features provides a useful evaluative framework, particularly in the absence of established experimental benchmarks.

The flexibility of the MOV&RSim algorithm enables its application across a wide range of scenarios. While primarily designed to generate DNA-seq data, the Editing Genome block can be used as a standalone tool to create mutated genomes, which can serve as input for RNA-seq simulators [44, 45] to produce transcriptomics NGS data. For instance, simulating variants near splicing sites, potentially linked to aberrant alternative splicing events in cancer transcriptomics, could be valuable for evaluating bioinformatics tools that process RNA-seq reads.

Future work will aim to further improve MOV&RSim by implementing additional simulation features, such as the integration of knowledgebase information to insert repeat-mediated variants, similar to what is done by SCNVSim. Furthermore, we plan to expand the mining of variants’ statistical information to a wider range of databases. This will enable us to inform all biological parameters of the simulator, including the remaining variant types, characteristics, the clonal VAFs, and cancer-specific clonal phylogenies not present in TCGA and COSMIC.

## Conclusions

Personalised medicine strategies have seen significant successes in tumour prevention, diagnosis, and the development of targeted therapies. For effective utilisation of targeted therapies, it is crucial to accurately detect somatic variants. Therefore, to improve the performance of somatic variant detection pipelines across diverse tumoral scenarios, a cancer-specific gold standard considering both the biological intricacies of tumoral genomes and technical constraints of NGS sequencing is essential.

In this work, after conducting an extensive analysis of nine somatic sample simulators, we developed MOV&RSim, a novel data-driven simulator that generates biologically and technically realistic tumoral samples for assessing the performance of somatic variant callers.

MOV&RSim is equipped with a guided procedure to define the number, zygosity, lengths, and positions per variant type in accordance with the biological information contained in cancer-specific presets derived from COSMIC and TCGA databases for 21 cancer types. The innovative approach of MOV&RSim focuses on learning the average biological characteristics typical of a specific cancer type (mutation burden, spatial distribution, structural variant prevalence, etc.) instead of reproducing known variants as they are (see VarSim’s approach [33]). With this information, it generates novel variants, whose characteristics are statistically consistent with those of the variants observed in real tumor genomes. Once these variants are used to mutate a template genome, MOV&RSim learns coverage distribution, sequencing error profiles, and base qualities from real samples, and use such technical information to generate realistic reads from the mutated genome.

Compared to existing simulators, MOV&RSim enables a more precise simulation of tumoral genomes. In particular, the user can simulate all variant types (SNP, INS, DEL, DUP, INV, TRA, Complex), and fully define, for each individual variant, all its characteristic, like length, content, position, zygosity. This feature enables users to derive samples with increasing levels of genomic complexity, containing mutations in challenging regions, overlapping events which are difficult to detect even with the most advanced variant detection algorithms, and a wide range of custom alterations, from short point mutations to large structural variants. These include both copy number-altering (long DEL, INS, DUP) and copy number-neutral events (INV), as well as genomic abnormalities such as whole-chromosome or even whole-genome aneuploidies. Depending on the specific mutational events users aim to detect, certain variant calling approaches may be more suitable than others. With MOV&RSim, users can introduce the variant types of interest, generate the corresponding reads, and evaluate which variant caller performs best on the simulated samples, with full knowledge of the true variant types, characteristics, positions, etc.

To the best of our knowledge, MOV&RSim is the only tool capable of learning data-driven information on characteristics of both variants and reads from real samples. Due to the lack of a comprehensive and high-quality dataset of cancer-specific gold-standard tumoral specimens, this exclusive feature of MOV&RSim constitutes an important resource for the validation and subsequent optimisation of variant calling pipelines, as the simulated samples can be designed specifically for each scenario.

## Implementation

MOV&RSim is implemented in R, Python, and C languages. It is freely available under the GNU GPLv3 licence on GitLab (https://gitlab.com/sysbiobig/movarsim), and a Docker image is provided to ease its deployment and enhance reproducibility. Code documentation and usage tutorial are available on the git repository.

**Fig. 5:**
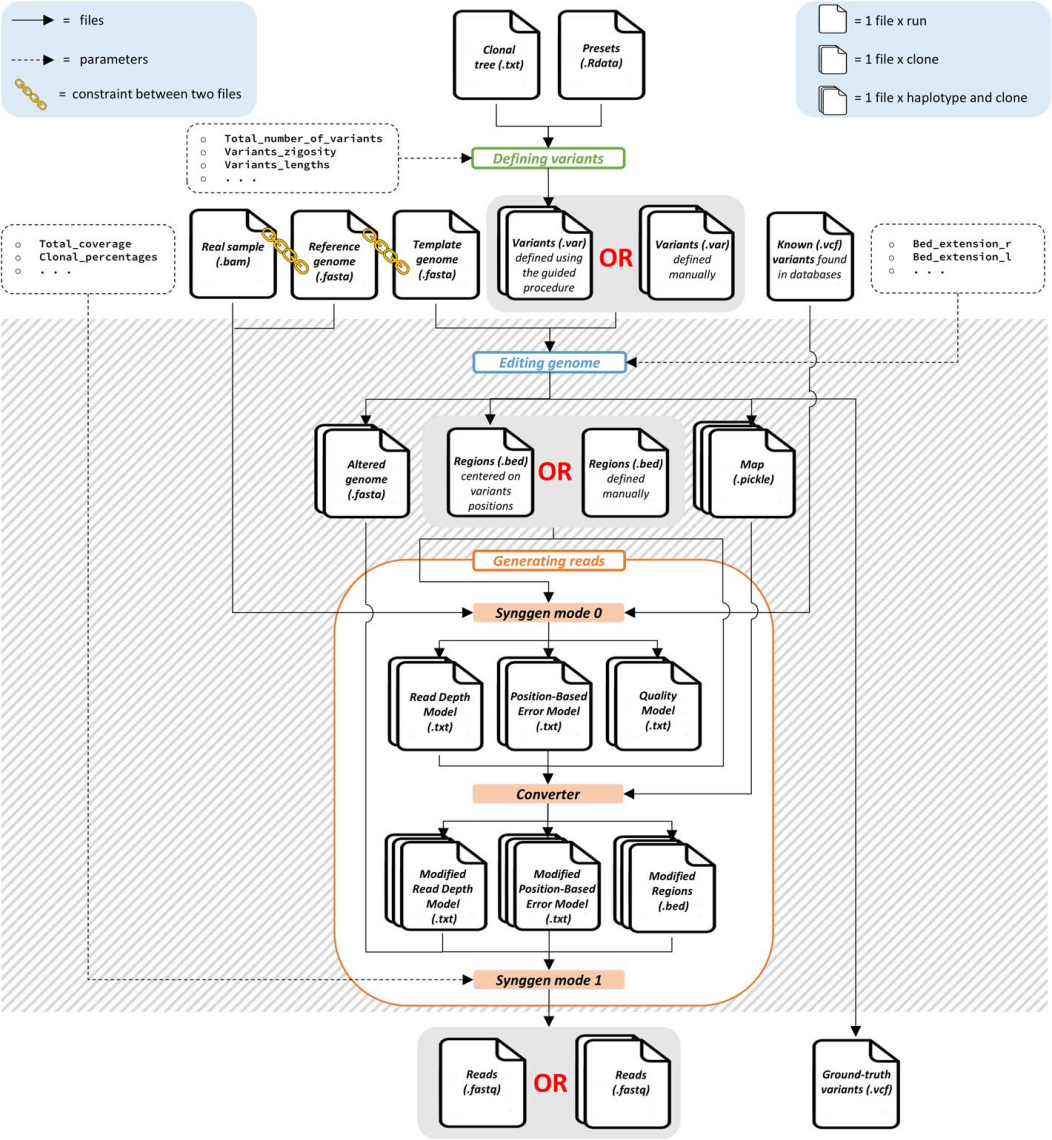
MOV&RSim simulation workflow, input/output files and formats. The MOV&RSim simulation workflow comprises three main blocks: Defining Variants, Editing Genome, and Generating Reads. The Generating Reads block is further divided into three sub-steps: Synggen mode 0, converter, and Synggen mode 1. Some files are designed to be shared across the entire workflow, others are specific to each clone, and some are specific to each haplotype of each clone. The chain symbol is used to indicate a constraint between two files. For instance, the BAM files of real samples used as input in Synggen mode 0 must have been aligned to the reference genome (FASTA format) that is also provided as input to Synggen mode 0. Additionally, the reference genome used for Synggen mode 0 must be equal to the template genome used in the Editing Genome block. The striped box indicates the mandatory part of the MOV&RSim simulation workflow. The figure includes a high level of detail aimed at showing all input, output, and intermediate files used by the simulator, but it is not necessary to run the workflow, as the management of multiple files related to different clones and their corresponding haplotypes is automated within the simulator

MOV&RSim can be divided into three main blocks according to their purpose (see Figs. 2 and 5): the first block (Defining variants) is optional and it can be used to define input variants, while the second (Editing genome) and third (Generating reads) blocks are mandatory and perform genome editing based on input variants and sequencing reads generation, respectively. Each block supports the use of dedicated random seeds to control stochasticity and ensure reproducibility across multiple runs.

### Editing genome block

#### Input

The input files for genome editing consist of a template genome in FASTA format and a list of variants that the user wishes to incorporate into the template genome. For the variant list, we created a format similar to the VAR, used in BAMSurgeon and SVEngine. Each line in the VAR format corresponds to an individual variant, allowing full control over all variant types and characteristics. In our implementation of the VAR format, the user specifies 9 values for each variant:

- VID (string), for univocal variant identification;
- MID (string), for tracking the same variant in different haplotypes or variants participating in complex events;
- HAP (integer), for the variant to alter the first haplotype (HAP = 0) or the second (HAP = 1);
- CHR (string), indicating the chromosome where the variant is located;
- POS (integer, 0-based), indicating the starting position of the variant;
- DEL (boolean), for specifying whether the variant comprises a DEL;
- DEL SPAN (integer), for specifying the DEL length (if DEL = True);
- INS (boolean), for specifying whether the variant comprises an INS;
- INS SEQ (string), for specifying the sequence of nucleotides to be inserted (if INS = True).

In order to define the INS SEQ, BAMSurgeon VAR format only accepts a sequence defined manually (e.g. “ACTG”). Differently, SVEngine VAR format requires the user to provide a genome file for extracting the INS SEQ, along with the chromosome and coordinates for the extraction, the number of times such sequence should be duplicated, and whether the sequence should be taken forward or reversed. However, BAMSurgeon and SVEngine formats lack the ability to define random nucleotide content events. To provide also this setting modality, the INS SEQ field in the MOV&RSim VAR format can be set to "random" followed by a number specifying the length of the desired random sequence. An example of MOV&RSim VAR file can be found in Fig. C2.

#### Variant simulation

**Fig. 6:**
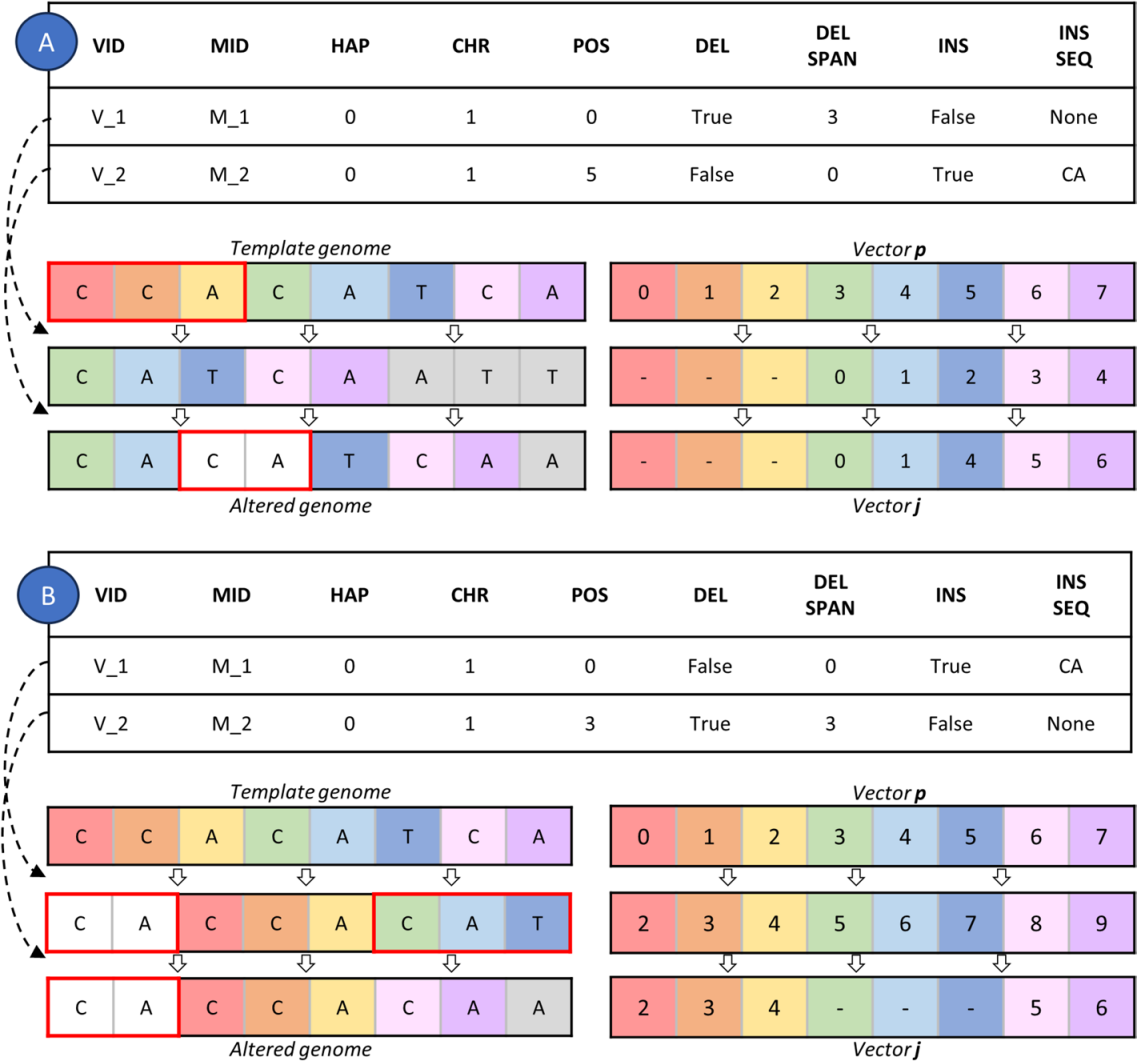
Variant incorporation strategy for 2 VAR files containing 2 variants. Under each VAR file it is showed how the template genome and the vector **j** (which initially equals the vector **p**) evolve after each variant incorporation. **A** DEL, V_1, in POS = 0 followed by INS, V_2, in POS = 5. Due to V_1, the nucleotide which originally was in POS = 5 maps in 2. Therefore, V_2 adds “CA” to the left of the nucleotide in 2. **B** INS, V_1, in POS = 0, followed by DEL, V_2, in POS = 3. Due to V_1, the nucleotide which originally was in POS = 3 maps in 5. Therefore, V_2 deletes 3 nucleotides located to the right of 5 (nucleotide in 5 included)

Since most simulators are limited in simulating overlapping variants (see Appendix A), we adopted a variant incorporation strategy that considers the order in which variants are specified in the VAR file (similar to the HeteroGenesis approach). Even if each variant’s position in the VAR file always refers to a position in the template genome, MOV&RSim incorporates the *i*-th variant in the VAR file into a version of the template genome resulting from the addition of the $(i-1)$-th variant, $(i-2)$-th variant, and so on up to the first variant in the VAR file. This was possible through the implementation of a map that univocally associates the position of each nucleotide in the template genome (where the user wishes to incorporate the variant) to its mapping position in the version of the template genome altered by previous variant incorporations (where the variant is actually incorporated) (Fig. 6).

Suppose the user wants to incorporate the first variant in the VAR file into the template genome, that contains *N* nucleotides. In the map, the positions of each nucleotide of the template genome are represented through the vector ${\textbf {p}} = 0, 1, \dots , N-1$. As no variant has been incorporated yet, mapping positions, indicated through the vector **j**, coincide with **p**. Using the VAR format, the user can incorporate either an INS or a DEL (combined events are split into DEL followed by INS):

- An INS in position $p(t) \in {\textbf {p}}$ adds an INS SEQ of $L_{ins}$ nucleotides to the left of the nucleotide in $j(p(t)) \in {\textbf {j}}$. After the INS, *j*(*p*(*t*)), $j(p(t+1))$, ..., $j(p(t+N-1))$ increase of $L_{ins}$.
- A DEL in position $p(t) \in {\textbf {p}}$ deletes the $L_{del}$ nucleotides located to the right of $j(p(t)) \in {\textbf {j}}$ (nucleotide in *j*(*p*(*t*)) included). After the DEL, *j*(*p*(*t*)), $j(p(t+1))$, ..., $j(p(t+L_{del}-1))$ are set to “–” and $j(p(t+L_{del}))$, $j(p(t+L_{del}+1))$, ..., $j(p(t+L_{del}+N-1))$ decrease of $L_{del}$.

Implementing the map allowed us to introduce another format for defining the INS SEQ in the VAR file. This format is similar to the one used for the SVEngine VAR format, with the exception that the genome from which the INS SEQ should be extracted is not specified, as the sequence is derived from the genome currently undergoing mutation. When using this format, the user needs only to specify a chromosome, an interval of **p**, e.g. from *j*(*p*(*k*)) to $j(p(k+L-1))$, the number of times such sequence should be duplicated, and whether it should be taken forward or reversed. This feature makes MOV&RSim the only simulator able to generate complex overlapping variants while accounting for all previously introduced variants in the genome sequence.

#### Output

The variant incorporation algorithm produces 4 types of output files: the altered genome sequence in FASTA, the map (stored with pickle [46]), ground truth variants in VCF, and a BED file containing coordinates of regions centered on the variant positions. These regions can be extended to the left and right as specified by the user and used in subsequent simulation steps. Positions in the VCF and BED files are indicated through **p** coordinates.

### Generating reads block

#### Input

The generating reads block takes as input the mutated genome (in FASTA format) and the map (in pickle format) produced as output in the previous block. Additionally, it requires the user to provide a list of regions of interest (BED format). The block also needs a list of real BAM files with technical characteristics (i.e. coverage, base quality, and sequencing error rates) that closely match those intended for simulation, together with the reference genome used for their alignment, and a list of real variants in VCF format (e.g. taken from dbSNP [47]).

#### Reads simulation

MOV&RSim adapts the Synggen workflow to generate reads from the input (altered) genome. First, it uses Synggen Mode 0, that takes as input the real BAM files, generated through any sequencing platform (Illumina, PacBio, etc.), the reference genome used for their alignment, and a BED file specifying the regions of interest. Synggen Mode 0 learns three error models that describe the technical characteristics of the aligned reads in the regions defined by the BED file: the Read Depth Model (RDM), which measures the depth of coverage in BED regions, the Quality Model (QM), which learns distribution of base qualities, and Position-Based Error Model (PBEM), which measures the probability of observing sequencing errors in each position of each BED region (excluding errors located within common SNPs positions, based on the provided VCF of real variants).

Next, Synggen Mode 1 takes as input the (altered) genome, the 3 errors models (RDM, QM, and PBEM) and the region of the genome where to simulate the reads (BED file) to generate a user-specified number reads in FASTQ format. In this way, the newly generated reads adopt the same technical characteristics as the real samples’ aligned reads, while containing the nucleotide content of the altered genome. Synggen operates only within the BED regions, allowing it to generate reads for WGS, WES, or TS, based on the coordinates provided in the BED file.

To allow MOV&RSim to leverage the information learned in Mode 0, while using Mode 1 for generating reads from the altered genome produced in the *Genome editing* step (rather than the template genome, as originally intended in Synggen’s original version), we developed an intermediate step, between Mode 0 and Mode 1, to adapt the BED file, RDM, and PBEM for each altered genome. As the BED coordinates and models’ information are using the coordinates of the template genome, i.e. the vector **p**, we implemented a converter that leverages on the map to switch them from the vector **p** to the corresponding vector **j**, that stores the mapping position of each nucleotide of the template genome in the altered genome:

- The BED file contains *p*(*start*) and *p*(*end*) of regions containing variants, or any other regions specified by the user. The converter creates a new BED file by mapping *p*(*start*) to *j*(*p*(*start*)) and *p*(*end*) to *j*(*p*(*end*)).
- The RDM is saved in a text file where each line corresponds to a BED region. Each line comprises 2 elements: the first is an integer that records the total number of BAM files’ reads having start alignment position inside the BED region, while the second is a vector of integers that records the number of aligned reads starting in each *p*(*t*) of that region. With these 2 elements, the RDM quantifies both the probability of observing reads within a given BED region and the probability of observing reads starting at any given position within the region. The converter re-calculates the second element, i.e. the vector of integers, saving the number of aligned reads starting in *p*(*t*), stored in the original RDM, to *j*(*p*(*t*)), in the new RDM. Then, it computes the first element, as the sum of all the integers in the new vector of integers.
- The PBEM is saved in a text file where each line corresponds to a BED region, and each line contains a list of six-number vectors to describe all the *p*(*t*) of the template genome which represent potential sequencing errors. Each six-number vector contains *p*(*t*), the number of reads found in *p*(*t*), and the cumulative number of As, Cs, Gs, and Ts found across the reads in *p*(*t*). The converter modifies each six-number vector by replacing *p*(*t*) with *j*(*p*(*t*)).

After the BED, RDM, and PBEM conversions, MOV&RSim can properly run Synggen Mode 1 on the altered genomes.

In terms of extension of the Synggen framework, we also enhanced the user options in terms of RDM and PBEM, adding the possibility to switch them off. This could be useful in benchmarking studies to assess which technical characteristics of the reads is affecting more the variant caller performance. To disable the RDM, we created a script that adjusts the RDM information in a way that each BED region and each position inside that region has an equal chance of being selected to initiate a read. To disable PBEM, we added a flag to the Synggen Mode 1 command that prevents the incorporation of sequencing errors into generated reads.

To simulate complex cancer scenarios, involving multiple clones, different levels of VAFs, strand bias effects, etc. while maintaining an easy-to-use tool for the user, we developed an ad-hoc procedure where the user can specify the total coverage for the final sample, the proportions of reads to be sampled from the first and second haplotypes, and the proportions of reads from each altered genome of the different clones. The procedure automatically computes the number of reads that MOV&RSim should generate from each haplotype (to represent heterozygous/homozygous variants and, eventually, the strand bias effect) of each altered genome (to represent the variants of different clones), and merges the generated reads into a unique final FASTQ file.

#### Output

The output of the generating reads block is a FASTQ files containing the simulated reads. In case of complex simulation scenarios involving multiple clones, MOV&RSim generates FASTQ files for each individual clone as well as a FASTQ file representing the final simulated sample. In the FASTQ file for the final simulated sample, all reads can be traced back to their originating clone through their identification string.

### Defining variants block

#### Cancer-specific presets: mining statistical information about variants from databases

To obtain data-driven information about variant characteristics in cancer-specific scenarios, we mined COSMIC and TCGA, focusing on the variant types available in both databases: SNPs, INSs, DELs, DUPs and DELINSs. When using COSMIC data, we considered variants found in genome-wide screened samples. For TCGA, we focused on variants detected in whole exome screened samples. From both databases, we obtained a list of known somatic variants. The two lists were then divided into 21 subsets each, corresponding to variants found in samples from distinct cancer types. We selected the 21 cancer types as those that are present and unambiguously named in both COSMIC and TCGA: breast, lung, colon, adrenal gland, biliary tract, bone, cervix, eye, kidney, liver, oesophagus, ovary, pancreas, pleura, prostate, skin, soft tissue, stomach, testis, thymus, thyroid. Data retrieval from COSMIC and TCGA is described in detail in Appendix B. We pre-processed the cancer-specific subsets from COSMIC and TCGA obtaining, for each variant, the information about the variant’s type, position, chromosome, and length (derived from the variant Human Genome Variation Society (HGVS) nomenclature). For DELINS, we derived separately the INS length component (delINS) and the DEL length component (DELins).

In each cancer-specific subset, we grouped all variants belonging to the same sample and we calculated the total number of variants per sample, as well as the proportions of SNPs, INSs, DELs, DUPs, and DELINSs and of homozygous/heterozygous variants (these latter in COSMIC subsets only, as the zygosity information is not available in TCGA) in each sample. For each variant type, we ordered variants by chromosome and by position. Then, we calculated the distances between adjacent variants in the same chromosome and, lastly, we aggregated all distances computed in all samples. These distributions can be sampled to sequentially derive realistic variant positions.

For each subset, we also calculated the overall proportions of different variant types and of homozygous/heterozygous variants across the entire subset, without aggregating variants by sample. In addition, we calculated variant lengths per variant type without aggregating variants by sample. As the SNP length is 0, and the length of DELINS depends on the delINS and the DELins, we focused on the following variant types for this computation: INSs, DELs, DUPs, delINS and DELins.

We processed all distributions by removing the occurrences with a relative frequency less than 1%. Lastly, we fitted the observed empirical distributions of the total number of variants per sample, variants lengths and distances per type to 10 known distributions, i.e. Weibull, lognormal, normal, negative binomial, exponential, logistic, Poisson, gamma, geometric, Cauchy, and evaluated the fits through 6 goodness-of-fit metrics, i.e. AIC, BIC, KS, AD, CvM, CHISQ. Note that for SNPs, theoretical distributions are fitted only against empirical distributions of SNPs positions, as SNPs lengths are represented by a uniform distribution with value 0 (DEL of 1 nucleotide, followed by INS of 1 nucleotide). For DELINS, theoretical distributions are fitted against the empirical distribution of the delINS and of the DELins. All fitting procedures were carried out using the R package *fitdistrplus*. It is important to point out that the presets are highly customisable and can be recalculated to represent the mutational profiles of specific cancer types and subtypes. By filtering the initial mutation database based on the desired simulation scenario (a particular tumor subtype, metastatic versus primary tissue, etc.), the corresponding distributions and proportions can be automatically generated. The scripts used to compute the presets from COSMIC and TCGA, cosmic_core_mutations.R and tgca_mutations.R, are available in the project repository at https://gitlab.com/sysbiobig/movarsim.

#### Implementing the guided procedure for defining variants

The process of manually defining variants to be included in the VAR file is error prone. The user can provide incorrect chromosome names (“chr1”, “1”, “chr_1”, $\ldots$), mention chromosomes that are not even present in the template genome, specify a position that goes outside the boundaries of the chromosome, etc. Therefore, to address these issues, we developed a guided procedure for compiling the VAR file. The procedure guides the user in setting the total number of variants in the VAR file, the proportion of heterozygous variants, the number of variants per type, and then, for each individual variant, its length and position. Once the setting is complete, the procedure shuffles all the variants listed in the VAR file to achieve a random insertion order for different variant types.

**Fig. 7:**
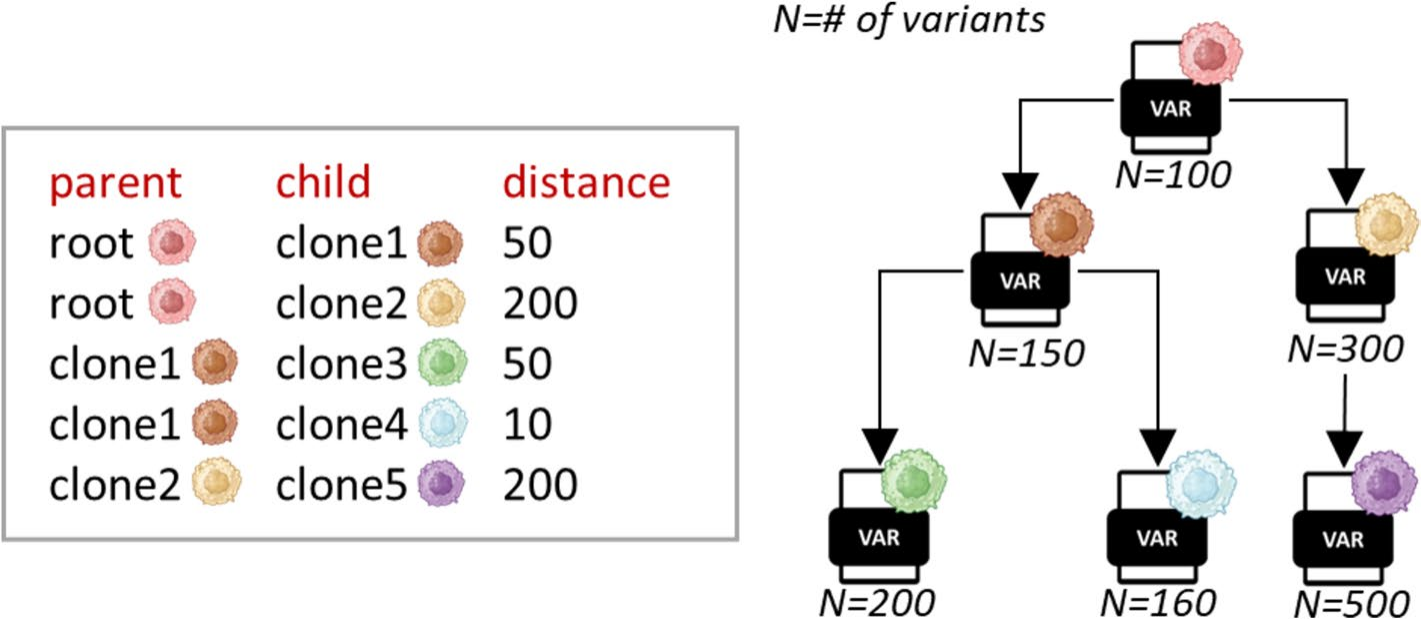
Construction of the VAR files representing each individual clone in the phylogeny. The format for specifying the clonal tree architecture is illustrated on the left. Each line corresponds to a branch of the clonal tree and it contains three values representing the parent clone, the child clone, and the evolutionary distance between them (i.e. number of variants acquired by the child from its parent). On the right, the graphical representation of the corresponding clonal tree. The number below each VAR file represents the total number of variants in the clone

Since the user may be interested in simulating several clones and defining their phylogenetic relationships, we made the procedure capable of accepting a clonal tree architecture and, starting from the initial VAR file representing the root clone, generating different versions of the original VAR file, one for each clone mentioned in the phylogeny (Fig. 7).

For the clonal tree architecture, we choose the HeteroGenesis format, as it provides the most precise framework for defining phylogenetic relationships between clones. This format consists of a text file where each line corresponds to a branch of the clonal tree. The user must specify three values representing, for each branch, the name of the parent clone, the name of its child, and the evolutionary distance between them, encoded in a number of variants acquired by the child that distinguish it from its parent. Therefore, when receiving a clonal tree architecture in this format, the procedure takes the parent VAR file for each branch, and adds the corresponding number of variants, guiding the user in defining their characteristics.

In addition, since the average user is often unaware of which variant characteristics should be selected to realistically simulate a biological sample, we followed the idea of tHapMix and allowed the user to set different variant characteristics according to data-driven proportions or distributions. When the user decides to follow this modality, the procedure integrates the presets we derived from COSMIC and TCGA, that contain information to describe the total number of variants, the number of variants per type, variants zygosity, lengths, and positions, in cancer-specific scenario.

Although the procedure is guided and user-friendly, it can take a long time to define interactively a large number of variants. Therefore, we gave the user the possibility to run the procedure automatically (instead of interactively) by asking the user the cancer type to simulate, the database on which to rely and, eventually, the total number of variants and the percentage of heterozygous variants.

### Case study settings

To demonstrate that MOV&RSim functions correctly, we simulated a toy sample containing few variants, including one example of each variant type that MOV&RSim can generate. To achieve this, we manually created a custom VAR file (*Defining variants* step) (see Fig. C2). We made all variants in homozigosity in order for them to appear in the reads with a Variant Allele Frequency (VAF) of 100%. Next, we executed MOV&RSim’s *Genome editing* step, employing GRCh38 as the template genome along with the custom VAR file. Following this, we executed the *Generating reads* step. We choose to generate paired-end reads, with a read length $L = 100$. To define the regions for read generation (i.e., regions where the first base of the first read in a pair could potentially start), we specified a BED file containing 400 bp regions centered on the simulated variants (total genome of interest $G \sim 4'400$ bp). We set the sequencing coverage to $C = 100\text {X}$. MOV&RSim automatically computes the number of reads to generate ($N_{reads}$) using the formula:

$$\begin{aligned} N_{reads} = \frac{G \times C}{L} \end{aligned}$$

Also, we disabled the RDM (equal probability for all BED regions to be selected for read generation and for all positions within a given BED region to be selected for initiating a read) and the PBEM (no sequencing errors). We aligned the generated reads using BWA-MEM2 [42] and visualised them with the IGV.

### Availability and requirements

- **Project name:** MOV&RSim
- **Project home page:** https://gitlab.com/sysbiobig/movarsim
- **Operating system:** Platform independent
- **Programming language:** R, Python, C
- **Other requirements:** see https://gitlab.com/sysbiobig/movarsim
- **License:** GNU GPLv3
- **Any restrictions to use by non-academics:** licence needed

## Supplementary Information

Supplementary Material 1.

## Data Availability

The data supporting the results of this article are available in the MOV&RSim repository, https://gitlab.com/sysbiobig/movarsim. The presets are also available at 10.5281/zenodo.11453104. Raw data for presets construction are available at 10.5281/zenodo.11458637.

## Abbreviations

NGS: Next generation sequencing
COSMIC: Catalogue of somatic mutations in cancer
TCGA: The cancer genome atlas
SNP: Single nucleotide polymorphism/s
INS: Insertion/s
DEL: Deletion/s
DUP: Duplication/s
TRA: Translocation/s
INV: Inversion/s
CNV: Copy number variant/s
VAF: Variant allele frequency
DELINS: Combined deletion/s–insertion/s
AIC: Akaike information criterion
BIC: Bayesian information criterion
KS: Kolmogorov–Smirnov
AD: Anderson–Darling
CvM: Cramer–von Mises
CHISQ: Chi square
delINS: INS length component
DELins: DEL length component
CDF: Cumulative density functions
bp: Base pairs
RDM: Read depth model
QM: Quality model
PBEM: Position-based error model
HGVS: Human genome variation society
WGS: Whole genome sequencing
WES: Whole exome sequencing
TS: Targeted sequencing
IGV: Integrative genomics viewer
TP: True positive
FP: False positive
FN: False negative
