# Supplementary material for: MOV&RSim: computational modelling of cancer-specific variants and sequencing reads characteristics for realistic tumoral sample simulation
Source: BMC Bioinformatics. 2025 Nov 27;26:287. doi: 10.1186/s12859-025-06292-0 (PMC12659228; doi:10.1186/s12859-025-06292-0)
Supplement: Supplementary file 1 — Supplementary Material 1. [file 12859_2025_6292_MOESM1_ESM.pdf]

MOV&RSim: computational modelling Of cancer-specific variants & sequencing reads characteristics for realistic tumoral sample simulation

Francesca Longhin[1], Giacomo Baruzzo[1*], Enidia Hazizaj[2], Diego Boscarino[2], Dino Paladin[2], Barbara Di Camillo[1,3,4*]

[1]Department of Information Engineering, University of Padova, Via Gradenigo 6b, Padova (PD), 35131, Veneto, Italy.
[2]AB ANALITICA, Srl, Via Svizzera 16, Padova (PD), 35127, Veneto, Italy.
[3]Department of Comparative Biomedicine and Food Science, University of Padova, Viale dell'Università 16, Legnaro (PD), 35020, Veneto, Italy.
[4]Padova Center of Network Medicine, Via Marzolo 8, Padova (PD), 35131, Veneto, Italy.

*Corresponding author(s). E-mail(s): giacomo.baruzzo@unipd.it; barbara.dicamillo@unipd.it;
Contributing authors: francesca.longhin.2@studenti.unipd.it; hazizaj@abanalitica.it; boscarino@abanalitica.it; paladin@abanalitica.it;

Appendix A Details about available simulators

In this section we evaluate 9 somatic sample simulators, namely Synggen, BAMSurgeon, SVEngine, VarSim, Xome-Blender, tHapMix, Pysim-sv, SCNVSim, and HeteroGenesis. We compared them in terms of simulation approach used to introduce variants, degree of user control over available parameters, considering both parameters that model the complexity of the tumoral genome (*biological parameters*), as well as the noise caused by library preparation and sequencing (*technical parameters*), and setting modalities (Figure 1, Table A1, Table A2). Additionally, we analised the different types of variants that can be introduced with each simulator: SNP, INS, DEL, DUP, INV, TRA, and complex overlapping variants. For DELs and DUPs, we attributed simulation capability to a given tool only if it could simulate both short ($<$ 50 nucleotides) and long events ($>$ 50 nucleotides), which results in CNV [1].

			Synggen	BAMSurgeon	SVEngine	VarSim	Xome-Blender	tHapMix	Pysim-sv	SCNVSim	HeteroGenesis	MOV&RSim
Biological Parameters	Variant	Number	●●● u	●●● u	●●● u	●●● db	●●● u	●●● u/db	●●● u	●● u	●● u	●●● u/D
		Position	●●● u	●●● u	●●● u	●●● db	●	●●● u/db	●●● db	●● u	●●● u/db	●●● u/D
		Length	●●● u	●●● u	●●● u	●●● db	●	●●● u/D	●●● u	●● u	●●● u/db	●●● u/D
		Content	●●● u	●●● u/∼	●●● u/db	●●● db	●	●●● u/db	●●● db/∼	●	●●● u/db/∼	●●● u/db/∼
		Zygosity	●●● u	●●● u	●●● u	●● u	●	●●● u/D	●●● u	●● u	●	●●● u/D/∼
	Sample	Clonality	●● af	●● af	●●● t	●	●● n	●●● t	●● n	●● n	●●● t	●●● t
		Purity	●● %	●● U	●● U	●● U	●● %	●● %	●● %	●● U	●● %	●● %/U
Technical Parameters	Reads	Coverage	●●● e	●● th	●● th	●● th	-	-	●● th	●● th	●● th	●●● e
		Base quality	●●● e	●● th	●● th	●●● e	-	-	●●● e	●●● e	●●● e	●●● e
		Sequencing errors	●●● e	●● th	●● th	●●● e	-	-	●●● e	●●● e	●●● e	●●● e

Table A1 Main features of 9 somatic sample simulators and MOV&RSim. User control: ●●● = high, ●● = medium, ● = none. Variant characteristics: user-defined (u), sampled from pre-compiled list (db), draw from distribution (D), random (∼). Sample clonality: defined through a tree topology (t), allele-frequency per variant (af), number of clones (n). Sample purity: defined through percentage (%) or by mixing the results of independent simulations (U). Reads characteristics: empirically derived (e) or theorical (th). The symbol "-" indicates that the simulator does not generate reads.

		Degree of user control	Setting modalities
Biological Parameters	Variant	High (•••) = Individual variant setting<br>Medium (••) = Rate or subset variant setting (e.g., one variant every 1000 bp)<br>None (•) = No control available	User-specified (u) = Characteristic defined by the user<br>Sample from pre-compiled list (db) = Characteristic defined by assigning the same value as a variant selected from a pre-compiled list (e.g., variant databases)<br>Draw from distribuion (D) = Characteristic defined by sampling the value from a data-driven distribution<br>Random (∼) = Characteristic defined by randomly sampling its value
	Sample	High (•••) = Relationships between cell populations can be defined both graphically and numerically<br>Medium (••) = Relationships between cell populations can be defined numerically (e.g. percentage of normal and tumoral tissue within the final simulated sample)<br>None (•) = No control available	Phylogenetic tree (t) = Clonality defined through a phylogenetic tree<br>Allele frequency (af) = Clonality defined by specifying the allele frequency with which each variant is incorporated into the reads<br>Number of clones (n) = Clonality defined by specifying the number of clones in the sample<br>Percentage (%) = Purity defined by specifying the percentage of contamination by healthy tissue<br>Union of independent simulations (U) = Purity defined by mixing reads generated through independent simulations
Technical Parameters	Reads	High (•••) = Control through a multi-parametric model (e.g. control coverage through specifying the total number of reads and their distribution over the genome)<br>Medium (••) = Control through a single-parameter model (e.g. control coverage through specifying the total number of reads)<br>None (•) = No control available	Empirical (e) = Characteristic described through an empirically derived model<br>Theorical (th) = Characteristic described through a theorical model

Table A2 Categories for defining the degree of user control and the available setting modalities in tumor sample simulators. User control is categorised into three levels (•••, ••, and •). There are four setting modalities for biological parameters that control variant characteristics (u, db, D, and ∼) and five for those controlling sample characteristics (t, af, n, %, and U). For technical parameters that control read characteristics, two categories are identified (e and th).

State-of-the-art simulators employ two primary simulation approaches to introduce mutations into synthetic samples: either by directly integrating mutations into a template DNA sequence and generating reads from the modified sequence (Synggen, SVEngine, VarSim, Pysim-sv, SCNVSim, HeteroGenesis), or by altering real sequencing data (Xome-Blender, tHapMix). BAMSurgeon uses both methods (the first for

short variants and the second for long variants). With the first approach, despite the ease of obtaining a reference sequence to serve as a template compared to obtaining real sequencing data, in-silico sequencing tends to approximate the inherent biases of real sequencing data [2]. As a consequence, simulated reads are much simpler and less noisy compared to real reads. Conversely, with the second approach, where existing sequencing reads are patched by the simulators, the need for in silico sequencing is bypassed. However, the simulation of variants is constrained by the fixed coverage of the inputted data.

Among the simulators falling into the first category, only Synggen was found to perform reads generation itself. In contrast, the remaining simulators rely on external read simulators, such as ART [3], Wessim [4], wgsim [5], or their adapted versions (w-Wessim [6], xwgsim [7], dwgsim [8]).

When compared to all other read simulators, Synggen provides the best framework for controlling read characteristics as it learns them all from empirical data through multi-parametric models. However, it does not manage INV and TRA. BAMSurgeon and SVEngine offer control over each variant characteristic for each variant type, leveraging the most flexible format to define variants, known as the VAR format. Nonetheless, BAMSurgeon implementation of tumoral clonality is not convenient as the user can only specify the fraction at which each variant is incorporated into the reads, without control over the clonal tree architecture. SVEngine partially overcomes this issue by computing the VAF of clonal variants based on a user-specified tree architecture. However, SVEngine requires the clonal tree architecture to be represented using a binary tree in which each internal node represents a population splitting variant. As a result, creating clones differentiated by a large number of variants, N, becomes cumbersome, as the user must manually design a binary tree with a height of $N-1$. Given that clones are often differentiated by a high number of variants [9] and that it is not provided an automatic procedure to derive the tree, SVEngine might

be unsuitable in many tumoral scenarios. The peculiarity of VarSim is that it samples lists of known mutations retrieved from databases. However, VarSim lacks the ability to simulate TRAs and does not provide any support for implementing sample clonality. Xome-Blender implements the so-called "morphological approach": it randomly removes variants from real sequencing reads, creating a new sample, progenitor of the initial sample. In this way, different clones can be simulated without introducing any synthetic element. Nevertheless, Xome-Blender lacks the ability to simulate the full spectrum of variant types. Also, variant characteristics cannot be customised as it relies on the variants already present in the initial sample. tHapMix is the only simulator that allows some variant characteristics to be set by sampling distributions learned from a list of known variants. This data-driven approach goes beyond the purely database-driven strategies of other tools, which simply spike in known variants found within existing databases. Yet, tHapMix is very limited in simulating different variant types. Pysim-sv has a specialised module for modelling GC-bias, which is one of the most prevalent biases in sequencing data, and SCNVSim offers a convenient framework for dealing with variants in repetitive regions. However, they do not provide control over the clonal tree architecture. Finally, several tools are available for simulating complex variants. BAMSurgeon and SVEngine, however, only simulate compound events (e.g., DELINS), whereas tHapMix, Pysim-sv, and Synggen simulate different variant types in separate stages (e.g., all small variants prior to CNV) [6]. HeteroGenesis stands out by sequentially incorporating different variant types in the order they are specified in the input file, providing the most efficient approach for creating overlapping events. Furthermore, it offers the most precise framework for defining the architecture of the clonal tree and characterising different clones by specifying the number of variants that differentiate each parent clone from its children. A drawback of Heterogenesis is the limited control over variant number, zygosity, and its inability to simulate TRAs.

Appendix B Details about the presets

We downloaded COSMIC Mutation Data (Genome Screens): https://cancer.sanger.ac.uk/cosmic/download/cosmic/v99/mutantcensus. To focus exclusively on somatic variants, we filtered the dataset retaining only the rows where the "MUTATION_SOMATIC_STATUS" column took the value "Confirmed somatic variant". This indicates that the mutation was confirmed as somatic through sequencing of both the tumor and a matched normal sample from the same patient. Then, in order to consider only WGS data, we retained the rows where "GENOME_WIDE_SCREEN" equals "y". To create the cancer-specific subsets, we sequentially filtered the whole dataset based on the "PRIMARY_SITE" column, selecting rows that matched any of the 21 specific cancer types: "breast", "lung", "colon", "adrenal_gland", "biliary_tract", "bone", "cervix", "eye", "kidney", "liver", "oesophagus", "ovary", "pancreas", "pleura", "prostate", "skin", "soft_tissue", "stomach", "testis", "thymus", "thyroid".

To access TCGA data, we used the GDC Data Portal: https://portal.gdc.cancer.gov/analysis_page?app=Downloads. We filtered the whole repository with the following options:

- **Data Category**: simple nucleotide variation;
- **Experimental Strategy**: WXS;
- **Data Format**: maf;
- **Access**: open.

At this stage, the repository contained MAF files for various samples, each containing aggregated and filtered WES mutation calls (i.e., Tier 3 data) from different variant calling pipelines (VarScan2 [10], Pindel [11], MuSe [12], MuTect2 [13]) applied to tumor-normal pairs. We further filtered the repository per cancer type (PRIMARY SITE should alternatively equal "breast", "lung", "colon", "adrenal gland", "bile duct", "bone", "cervix", "eye", "kidney", "liver", "esophagus", "ovary", "pancreas", "pleura",

“prostate”, “skin”, “soft tissue”, “stomach”, “testis”, “thymus”, “thyroid”). Finally, for each cancer type, we downloaded the MAF files corresponding to different samples and concatenated them into a single file.

The information contained in COSMIC and TCGA presets is summarised in the following tables. Table B3 reports the total number of samples in both databases, alongside the cumulative count of variants across all samples. Table B4 shows the proportions of each variant type in each database, with variants classified into SNPs, INSs, DELs, DUPs, and DELINS. For the COSMIC database only (as TCGA does not provide this type of information), Table B5 displays the proportions of heterozygous and homozygous variants. Lastly, Tables B6-B11 reports the best fitting theoretical distributions, according to the minimisation of different goodness-of-fit metrics (AIC, BIC, KS, AD, CvM, CHISQ, respectively) for representing the total number of variants, variant lengths, and the positions per variant type in each database. Table B12 reports the best fitting theoretical distributions based on majority voting across the different goodness-of-fit metrics.

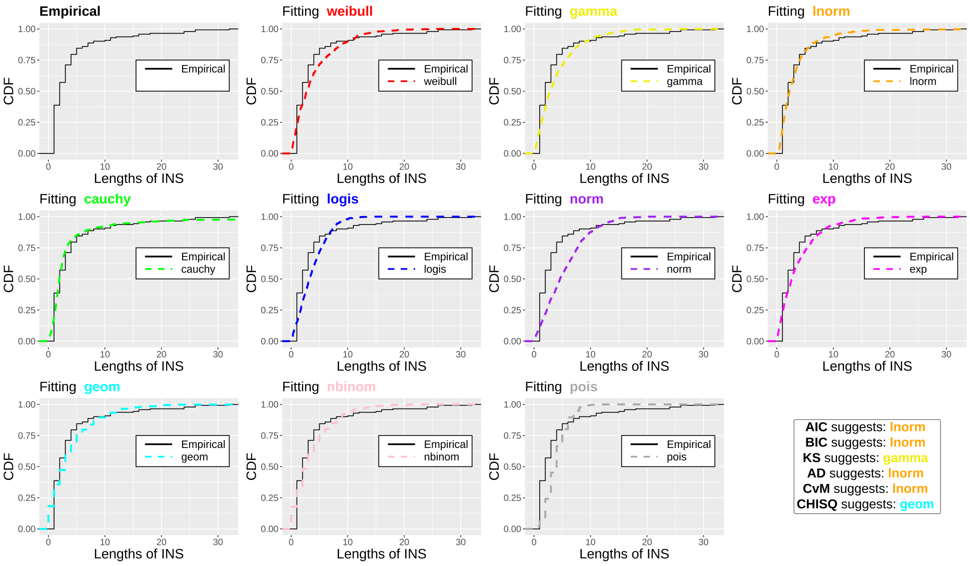

Fig. B1 Visualisation to guide users deciding which distribution to sample for each variant characteristic and type. In this case, the variant characteristic is the length, while the variant type is INSs. This kind of visualisation is only shown to the users when they decide to use the procedure interactively to set a certain variant characteristic using the distribution-driven approach.

Database	Cancer Type	Samples	Variants
COSMIC	Breast	12’423	1’303’458
	Lung	20’019	1’056’905
	Colon	42’949	1’509’657
	Adrenal Gland	1’938	8’235
	Biliary Tract	2’949	436’992
	Bone	1’852	43’463
	Cervix	1’040	88’240
	Eye	1’299	4’447
	Kidney	6’201	333’005
	Liver	4’721	2’039’690
	Oesophagus	3’757	877’027
	Ovary	5’575	467’934
	Pancreas	8’961	1’123’268
	Pleura	567	5’717
	Prostate	4’401	903’835
	Skin	14’161	1’723’754
	Soft Tissue	8’488	352’127
	Stomach	4’213	847’375
	Testis	489	4’219
	Thymus	184	1’543
	Thyroid	18’429	206’299
TCGA	Breast	1’384	121’762
	Lung	1’566	506’125
	Colon	650	361’124
	Adrenal Gland	435	12’158
	Biliary Tract	23	1’847
	Bone	152	5’092
	Cervix	374	74’906
	Eye	80	1’489
	Kidney	1’180	76’114
	Liver	419	53’646
	Oesophagus	236	38’849
	Ovary	537	69’555
	Pancreas	536	46’664
	Pleura	135	3’539
	Prostate	554	27’785
	Skin	522	408’555
	Soft Tissue	136	12’741
	Stomach	480	204’015
	Testis	130	2’664
	Thymus	91	1’980
	Thyroid	498	6’304

Table B3 Total number of samples, along with their cumulative number of variants, for 21 distinct cancer types in COSMIC and TCGA.

Database	Cancer Type	SNP	INS	DEL	DUP	DELINS
COSMIC	Breast	0.9453	0.0038	0.0348	0.0153	0.0008
	Lung	0.9742	0.0027	0.0134	0.0049	0.0048
	Colon	0.8953	0.0021	0.0794	0.0224	0.0008
	Adrenal Gland	0.9308	0.0079	0.0484	0.0059	0.0070
	Biliary Tract	0.4081	0.0333	0.2911	0.2673	0.0002
	Bone	0.8812	0.0067	0.0844	0.0266	0.0011
	Cervix	0.9926	0.0008	0.0015	0.0045	0.0006
	Eye	0.8604	0.0319	0.0760	0.0106	0.0211
	Kidney	0.9709	0.0016	0.0211	0.0054	0.0009
	Liver	0.9698	0.0017	0.0203	0.0082	0.0000
	Oesophagus	0.9684	0.0049	0.0165	0.0102	0.0000
	Ovary	0.9877	0.0009	0.0063	0.0047	0.0004
	Pancreas	0.9851	0.0009	0.0083	0.0053	0.0001
	Pleura	0.9038	0.0032	0.0744	0.0173	0.0013
	Prostate	0.8413	0.0129	0.0737	0.0702	0.0018
	Skin	0.9693	0.0035	0.0083	0.0020	0.0168
	Soft Tissue	0.9439	0.0048	0.0205	0.0303	0.0005
	Stomach	0.8699	0.0040	0.0944	0.0251	0.0065
	Testis	0.9351	0.0042	0.0456	0.0114	0.0037
	Thymus	0.9074	0.0136	0.0557	0.0226	0.0006
	Thyroid	0.9769	0.0031	0.0139	0.0057	0.0004
TCGA	Breast	0.9423	0.0012	0.0449	0.0102	0.0014
	Lung	0.9673	0.0011	0.0249	0.0058	0.0009
	Colon	0.8868	0.0007	0.0901	0.0219	0.0005
	Adrenal Gland	0.9377	0.0009	0.0488	0.0116	0.0010
	Biliary Tract	0.9616	0.0010	0.0222	0.0136	0.0016
	Bone	0.9513	0.0009	0.0315	0.0148	0.0015
	Cervix	0.9709	0.0005	0.0221	0.0060	0.0004
	Eye	0.9557	0.0006	0.0329	0.0094	0.0013
	Kidney	0.8845	0.0037	0.0878	0.0204	0.0035
	Liver	0.8839	0.0013	0.0844	0.0290	0.0014
	Oesophagus	0.9369	0.0012	0.0464	0.0146	0.0008
	Ovary	0.9442	0.0024	0.0336	0.0185	0.0012
	Pancreas	0.9637	0.0012	0.0236	0.0110	0.0005
	Pleura	0.9311	0.0016	0.0529	0.0116	0.0028
	Prostate	0.9408	0.0014	0.0447	0.0121	0.0010
	Skin	0.9937	0.0002	0.0045	0.0011	0.0004
	Soft Tissue	0.9657	0.0005	0.0269	0.0055	0.0011
	Stomach	0.8487	0.0007	0.1246	0.0254	0.0005
	Testis	0.9276	0.0015	0.0619	0.0082	0.0007
	Thymus	0.8773	0.0015	0.1005	0.0196	0.0010
	Thyroid	0.9521	0.0014	0.0365	0.0094	0.0006

Table B4 The proportions of SNPs, INSs, DELs, DUPs, and DELINSs among all variants in COSMIC and TCGA for 21 distinct cancer types.

Database	Cancer Type	Heterozygous	Homozygous
COSMIC	Breast	0.9561	0.0439
	Lung	0.9475	0.0525
	Colon	0.9368	0.0632
	Adrenal Gland	0.9504	0.0496
	Biliary Tract	0.9796	0.0204
	Bone	0.9040	0.0960
	Cervix	0.5882	0.4118
	Eye	0.8672	0.1328
	Kidney	0.4895	0.5105
	Liver	0.9945	0.0055
	Oesophagus	0.9559	0.0441
	Ovary	0.9565	0.0435
	Pancreas	0.9552	0.0448
	Pleura	0.9592	0.0408
	Prostate	0.9865	0.0135
	Skin	0.9282	0.0718
	Soft Tissue	0.9034	0.0966
	Stomach	0.9849	0.0151
	Testis	0.9967	0.0033
	Thymus	0.9592	0.0408
	Thyroid	0.9851	0.0149

Table B5 The proportions of heterozygous and homozygous variants, among all variants in COSMIC, for 21 distinct cancer types.

Database	Cancer Type	Total number of variants	SNP	INS		DEL		DUP		DELINS		
			Position	Length	Position	Length	Position	Length	Position	Length (INS)	Length (DEL)	Position
**COSMIC**	Breast	LN	W	LN	NB	LN	NB	L	NB	LN	LN	NB
	Lung	LN	NB	LN	NB	LN	NB	LN	NB	L	LN	NB
	Colon	LN	W	LN	NB	LN	W	L	NB	LN	LN	NB
	Adrenal Gland	LN	NB	G	NB	LN	NB	LN	W	LN	LN	NB
	Biliary Tract	LN	W	LN	NB	LN	W	LN	W	LN	LN	W
	Bone	LN	NB	LN	NB	LN	NB	LN	NB	LN	LN	NB
	Cervix	LN	NB	LN	NB	LN	W	LN	NB	LN	LN	NB
	Eye	LN	NB	LN	NB	LN	NB	LN	W	LN	LN	NB
	Kidney	LN	W	LN	NB	LN	NB	LN	NB	L	LN	NB
	Liver	LN	W	LN	NB	LN	NB	LN	NB	LN	LN	NB
	Oesophagus	LN	W	LN	NB	LN	NB	L	NB	LN	LN	LN
	Ovary	LN	W	LN	NB	LN	NB	LN	NB	L	LN	W
	Pancreas	LN	W	LN	NB	LN	NB	LN	NB	LN	LN	NB
	Pleura	LN	NB	LN	-	LN	NB	LN	LN	LN	E	-
	Prostate	LN	W	LN	NB	LN	NB	LN	W	LN	W	NB
	Skin	LN	W	LN	NB	LN	NB	LN	NB	N	E	NB
	Soft Tissue	LN	W	LN	NB	LN	NB	LN	W	LN	LN	NB
	Stomach	LN	W	LN	NB	LN	W	L	NB	LN	LN	LN
	Testis	LN	NB	LN	P	LN	LN	LN	LN	L	LN	-
	Thymus	LN	NB	LN	NB	LN	W	LN	N	-	-	-
	Thyroid	LN	W	LN	NB	LN	NB	LN	NB	LN	LN	-
**TCGA**	Breast	LN	NB	LN	NB	LN	NB	P	NB	LN	G	NB
	Lung	G	NB	LN	W	LN	NB	P	NB	LN	LN	W
	Colon	C	W	LN	NB	LN	NB	P	NB	E	W	NB
	Adrenal Gland	LN	NB	LN	NB	LN	NB	P	NB	LN	C	LN
	Biliary Tract	N	NB	E	-	LN	W	P	W	P	P	-
	Bone	G	NB	LN	-	LN	W	P	W	LN	E	-
	Cervix	LN	NB	LN	NB	LN	NB	P	NB	LN	LN	W
	Eye	C	NB	-	-	LN	LN	P	-	W	W	-
	Kidney	W	NB	LN	NB	LN	NB	P	NB	LN	G	LN
	Liver	LN	NB	LN	W	LN	W	P	W	LN	G	-
	Oesophagus	NB	NB	LN	-	LN	W	P	NB	LN	E	-
	Ovary	NB	NB	LN	NB	LN	NB	P	NB	LN	G	C
	Pancreas	W	NB	LN	NB	LN	NB	P	NB	LN	LN	-
	Pleura	W	NB	LN	-	LN	NB	P	N	LN	W	-
	Prostate	NB	NB	LN	NB	LN	NB	P	NB	LN	LN	-
	Skin	W	NB	LN	LN	LN	NB	P	NB	LN	C	LN
	Soft Tissue	LN	NB	LN	-	LN	NB	P	LN	C	LN	-
	Stomach	LN	W	LN	W	LN	NB	P	NB	LN	LN	W
	Testis	LN	NB	LN	-	LN	NB	P	-	W	P	-
	Thymus	LN	NB	P	-	LN	W	P	LN	P	E	-
	Thyroid	LN	NB	LN	-	LN	NB	P	N	LN	P	-

Table B6 The optimal theoretical distributions, based on the AIC metric, for modeling the total number of variants, as well as the lengths and positions of SNPs, INSs, DELs, DUPs, and DELINSs in samples of 21 cancer types from COSMIC and TCGA. Weibull = W, Gamma = G, Lognormal = LN, Cauchy = C, Logistic = L, Normal = N, Exponential = E, Geometric = GE, negative binomial =NB, Poisson = P. The symbol "-" means that all fits failed.

Database	Cancer Type	Total number of variants	SNP	INS		DEL		DUP		DELINS		
			Position	Length	Position	Length	Position	Length	Position	Length (INS)	Length (DEL)	Position
COSMIC	Breast	LN	W	LN	NB	LN	NB	L	NB	LN	LN	NB
	Lung	LN	NB	LN	NB	LN	NB	LN	NB	L	LN	NB
	Colon	LN	W	LN	NB	LN	W	L	NB	LN	LN	NB
	Adrenal Gland	LN	NB	G	NB	LN	NB	LN	W	LN	LN	NB
	Biliary Tract	LN	W	LN	NB	LN	W	LN	W	LN	LN	W
	Bone	LN	NB	LN	NB	LN	NB	LN	NB	LN	LN	NB
	Cervix	LN	NB	LN	NB	LN	W	LN	NB	LN	LN	NB
	Eye	LN	NB	LN	NB	LN	NB	LN	W	LN	LN	NB
	Kidney	LN	W	LN	NB	LN	NB	LN	NB	L	LN	NB
	Liver	LN	W	LN	NB	LN	NB	LN	NB	LN	LN	NB
	Oesophagus	LN	W	LN	NB	LN	NB	L	NB	LN	LN	LN
	Ovary	LN	W	LN	NB	LN	NB	LN	NB	L	LN	W
	Pancreas	LN	W	LN	NB	LN	NB	LN	NB	LN	LN	NB
	Pleura	LN	NB	LN	-	LN	NB	LN	LN	LN	E	-
	Prostate	LN	W	LN	NB	LN	NB	LN	W	LN	W	NB
	Skin	LN	W	LN	NB	LN	NB	LN	NB	N	E	NB
	Soft Tissue	LN	W	LN	NB	LN	NB	LN	W	LN	LN	NB
	Stomach	LN	W	LN	NB	LN	W	L	NB	LN	LN	LN
	Testis	LN	NB	LN	P	LN	LN	LN	LN	L	LN	-
	Thymus	LN	NB	LN	NB	LN	W	LN	N	-	-	-
	Thyroid	LN	W	LN	NB	LN	NB	LN	NB	LN	LN	-
TCGA	Breast	LN	NB	LN	NB	LN	NB	P	NB	LN	G	NB
	Lung	G	NB	LN	W	LN	NB	P	NB	LN	LN	W
	Colon	C	W	LN	NB	LN	NB	P	NB	E	W	NB
	Adrenal Gland	LN	NB	LN	NB	LN	NB	P	NB	LN	C	LN
	Biliary Tract	N	NB	P	-	LN	W	P	W	P	P	-
	Bone	E	NB	LN	-	LN	W	P	W	LN	E	-
	Cervix	LN	NB	LN	NB	LN	NB	P	NB	LN	LN	W
	Eye	C	NB	-	-	LN	LN	P	-	W	W	-
	Kidney	W	NB	LN	NB	LN	NB	P	NB	LN	G	LN
	Liver	LN	NB	LN	W	LN	W	P	W	LN	G	-
	Oesophagus	NB	NB	LN	-	LN	W	P	NB	LN	E	-
	Ovary	NB	NB	LN	NB	LN	NB	P	NB	LN	G	C
	Pancreas	W	NB	LN	NB	LN	NB	P	NB	LN	LN	-
	Pleura	E	NB	LN	-	LN	NB	P	N	LN	W	-
	Prostate	NB	NB	LN	NB	LN	NB	P	NB	LN	LN	-
	Skin	W	NB	LN	LN	LN	NB	P	NB	LN	C	LN
	Soft Tissue	LN	NB	LN	-	LN	NB	P	LN	C	LN	-
	Stomach	LN	W	LN	W	LN	NB	P	NB	LN	LN	W
	Testis	LN	NB	LN	-	LN	NB	P	-	W	P	-
	Thymus	LN	NB	P	-	LN	W	P	LN	P	E	-
	Thyroid	LN	NB	LN	-	LN	NB	P	N	LN	P	-

Table B7 The optimal theoretical distributions, based on the BIC metric, for modeling the total number of variants, as well as the lengths and positions of SNPs, INSs, DELs, DUPs, and DELINSs in samples of 21 cancer types from COSMIC and TCGA. Weibull = W, Gamma = G, Lognormal = LN, Cauchy = C, Logistic = L, Normal = N, Exponential = E, Geometric = GE, negative binomial = NB, Poisson = P. The symbol "-" means that all fits failed.

Database	Cancer Type	Total number of variants	SNP	INS		DEL		DUP		DELINS		
			Position	Length	Position	Length	Position	Length	Position	Length (INS)	Length (DEL)	Position
**COSMIC**	Breast	LN	W	LN	N	W	W	L	W	LN	LN	W
	Lung	W	W	LN	W	W	W	L	W	L	L	W
	Colon	W	W	G	W	W	W	L	NB	LN	LN	W
	Adrenal Gland	L	W	L	L	W	W	W	C	L	L	W
	Biliary Tract	LN	W	LN	W	L	W	L	W	W	W	LN
	Bone	W	W	G	N	W	W	L	W	E	W	W
	Cervix	W	W	LN	L	W	LN	L	W	L	L	W
	Eye	W	W	LN	W	W	N	N	W	L	W	W
	Kidney	LN	W	G	L	W	W	L	W	G	L	W
	Liver	W	W	LN	LN	L	W	L	W	LN	W	L
	Oesophagus	LN	LN	LN	W	N	W	L	W	L	L	LN
	Ovary	W	W	E	W	W	W	L	W	W	W	W
	Pancreas	W	W	G	N	N	W	L	W	LN	W	W
	Pleura	W	W	LN	-	W	W	L	LN	L	L	-
	Prostate	LN	W	LN	W	N	W	L	W	W	W	N
	Skin	W	W	W	W	W	W	L	W	LN	E	W
	Soft Tissue	W	W	LN	W	W	W	L	W	LN	W	L
	Stomach	LN	W	LN	W	W	W	L	W	L	L	LN
	Testis	LN	LN	L	-	G	LN	N	LN	L	L	-
	Thymus	LN	W	LN	C	G	LN	L	L	-	-	-
	Thyroid	L	W	LN	N	W	W	W	W	LN	W	-
**TCGA**	Breast	LN	W	G	L	W	W	E	W	LN	W	L
	Lung	G	W	N	W	W	W	E	W	W	LN	W
	Colon	C	W	L	W	L	W	E	W	G	W	C
	Adrenal Gland	LN	W	W	C	W	W	E	W	L	C	C
	Biliary Tract	N	LN	E	-	W	N	E	C	L	L	-
	Bone	G	W	L	-	G	L	E	W	LN	E	-
	Cervix	LN	W	E	C	N	W	E	W	C	LN	C
	Eye	C	W	-	-	W	LN	E	-	C	C	-
	Kidney	N	W	G	L	N	W	E	W	W	LN	LN
	Liver	LN	W	W	L	L	W	E	LN	G	W	-
	Oesophagus	G	W	L	-	W	W	E	W	E	E	-
	Ovary	G	W	L	W	G	W	E	W	G	W	C
	Pancreas	L	W	G	LN	W	W	E	W	LN	G	-
	Pleura	L	W	LN	-	W	W	E	C	L	C	-
	Prostate	L	W	LN	C	W	W	E	W	LN	LN	-
	Skin	W	W	G	L	W	W	E	W	L	L	W
	Soft Tissue	C	W	L	-	W	W	E	W	C	W	-
	Stomach	LN	W	W	W	W	W	E	W	W	LN	L
	Testis	LN	N	L	-	LN	W	E	-	C	C	-
	Thymus	LN	W	E	-	W	W	E	LN	C	C	-
	Thyroid	LN	W	L	-	W	W	E	C	L	L	-

Table B8 The optimal theoretical distributions, based on the KS metric, for modeling the total number of variants, as well as the lengths and positions of SNPs, INSs, DELs, DUPs, and DELINSs in samples of 21 cancer types from COSMIC and TCGA. Weibull = W, Gamma = G, Lognormal = LN, Cauchy = C, Logistic = L, Normal = N, Exponential = E, Geometric = GE, negative binomial = NB, Poisson = P. The symbol "-" means that all fits failed.

Database	Cancer Type	Total number of variants	SNP	INS		DEL		DUP		DELINS		
			Position	Length	Position	Length	Position	Length	Position	Length (INS)	Length (DEL)	Position
COSMIC	Breast	LN	W	LN	L	W	W	W	W	LN	LN	W
	Lung	LN	W	LN	L	W	L	W	W	W	W	W
	Colon	W	W	LN	L	W	W	E	W	LN	LN	L
	Adrenal Gland	W	W	N	L	LN	W	W	C	LN	W	W
	Biliary Tract	LN	W	LN	W	W	W	E	W	LN	W	W
	Bone	W	W	LN	L	LN	W	W	W	LN	W	W
	Cervix	W	W	LN	L	W	W	W	W	LN	W	W
	Eye	W	W	LN	W	LN	L	E	W	LN	LN	W
	Kidney	LN	W	LN	L	W	W	W	W	G	E	W
	Liver	W	W	LN	L	W	W	W	W	LN	W	L
	Oesophagus	W	LN	LN	W	W	W	W	W	LN	W	LN
	Ovary	W	W	LN	W	W	W	W	W	W	W	W
	Pancreas	LN	W	LN	L	W	W	W	W	LN	E	W
	Pleura	LN	W	LN	-	W	L	E	LN	G	L	-
	Prostate	LN	W	LN	W	W	W	W	W	LN	W	L
	Skin	W	W	LN	W	W	W	W	W	LN	E	W
	Soft Tissue	W	W	LN	L	W	W	W	W	LN	W	L
	Stomach	W	W	LN	W	W	W	W	W	W	W	W
	Testis	LN	L	W	-	LN	LN	L	LN	LN	W	-
	Thymus	LN	L	LN	C	LN	W	W	C	-	-	-
	Thyroid	W	W	LN	L	LN	W	W	W	LN	W	-
TCGA	Breast	LN	W	LN	L	W	W	E	W	LN	G	L
	Lung	G	W	W	W	W	W	E	W	LN	LN	W
	Colon	LN	W	W	W	W	W	E	W	G	W	C
	Adrenal Gland	LN	W	E	C	LN	W	E	W	W	LN	LN
	Biliary Tract	N	LN	E	-	LN	L	E	C	W	W	-
	Bone	G	W	W	-	LN	L	E	W	LN	E	-
	Cervix	LN	W	LN	C	W	W	E	W	W	LN	C
	Eye	LN	W	-	-	W	LN	E	-	C	C	-
	Kidney	N	W	LN	L	W	W	E	W	LN	G	LN
	Liver	LN	W	LN	L	W	W	E	W	LN	G	-
	Oesophagus	G	W	W	-	W	W	E	W	LN	E	-
	Ovary	G	W	W	W	G	W	E	W	LN	W	LN
	Pancreas	L	W	LN	L	LN	W	E	W	LN	LN	-
	Pleura	L	W	LN	-	W	W	E	C	W	L	-
	Prostate	L	W	LN	C	W	W	E	W	LN	LN	-
	Skin	W	W	LN	L	W	W	E	W	L	LN	LN
	Soft Tissue	C	W	G	-	LN	W	E	LN	C	LN	-
	Stomach	LN	W	W	W	W	W	E	W	LN	LN	L
	Testis	LN	L	W	-	LN	W	E	-	C	C	-
	Thymus	LN	W	E	-	W	W	E	LN	C	C	-
	Thyroid	LN	W	L	-	LN	L	E	C	W	L	-

Table B9 The optimal theoretical distributions, based on the AD metric, for modeling the total number of variants, as well as the lengths and positions of SNPs, INSs, DELs, DUPs, and DELINSs in samples of 21 cancer types from COSMIC and TCGA. Weibull = W, Gamma = G, Lognormal = LN, Cauchy = C, Logistic = L, Normal = N, Exponential = E, Geometric = GE, negative binomial = NB, Poisson = P. The symbol "-" means that all fits failed.

Database	Cancer Type	Total number of variants	SNP	INS		DEL		DUP		DELINS		
			Position	Length	Position	Length	Position	Length	Position	Length (INS)	Length (DEL)	Position
COSMIC	Breast	LN	W	LN	L	LN	W	L	W	LN	LN	W
	Lung	LN	W	LN	W	W	W	L	W	G	GE	W
	Colon	LN	W	LN	L	L	W	L	W	LN	LN	W
	Adrenal Gland	W	W	N	L	LN	W	LN	C	L	L	W
	Biliary Tract	LN	W	LN	W	L	W	L	W	LN	LN	W
	Bone	W	W	LN	L	LN	W	L	W	LN	LN	W
	Cervix	LN	W	LN	L	W	LN	L	W	L	L	W
	Eye	LN	W	LN	W	LN	W	L	W	LN	L	W
	Kidney	LN	W	LN	L	LN	W	L	W	G	L	W
	Liver	W	W	LN	L	L	W	L	W	LN	W	N
	Oesophagus	W	LN	LN	W	W	W	L	W	L	L	LN
	Ovary	LN	W	LN	W	LN	W	L	W	L	L	W
	Pancreas	LN	W	LN	L	W	W	L	W	LN	L	W
	Pleura	LN	W	LN	-	W	L	L	LN	L	L	-
	Prostate	LN	W	LN	W	W	W	L	W	LN	W	L
	Skin	W	W	LN	W	LN	W	L	W	LN	E	W
	Soft Tissue	LN	W	LN	L	LN	W	L	W	LN	W	L
	Stomach	W	W	LN	W	L	W	L	W	W	W	W
	Testis	LN	L	L	-	LN	LN	L	LN	LN	L	-
	Thymus	LN	L	LN	C	LN	W	L	C	-	-	-
	Thyroid	L	W	LN	L	LN	W	LN	W	LN	W	-
TCGA	Breast	LN	W	LN	L	LN	W	E	W	LN	G	L
	Lung	G	W	L	W	W	W	E	W	L	LN	W
	Colon	C	W	L	W	L	W	E	W	G	W	C
	Adrenal Gland	LN	W	LN	C	LN	W	E	W	L	LN	LN
	Biliary Tract	N	L	E	-	LN	L	E	C	L	L	-
	Bone	G	W	L	-	LN	L	E	W	LN	E	-
	Cervix	LN	W	LN	C	LN	W	E	W	L	LN	C
	Eye	LN	W	-	-	W	LN	E	-	C	C	-
	Kidney	N	W	LN	L	W	W	E	W	L	LN	LN
	Liver	LN	W	LN	L	W	W	E	LN	LN	G	-
	Oesophagus	G	W	W	-	LN	W	E	W	LN	E	-
	Ovary	G	W	L	W	W	W	E	W	LN	E	C
	Pancreas	L	LN	LN	W	LN	W	E	W	LN	LN	-
	Pleura	L	W	LN	-	LN	W	E	C	L	C	-
	Prostate	L	W	LN	C	LN	W	E	W	LN	LN	-
	Skin	W	W	LN	L	LN	W	E	W	L	C	LN
	Soft Tissue	C	W	L	-	LN	W	E	LN	C	LN	-
	Stomach	LN	W	L	L	L	W	E	W	LN	LN	L
	Testis	LN	L	L	-	LN	L	E	-	C	C	-
	Thymus	LN	W	E	-	L	W	E	LN	C	C	-
	Thyroid	LN	W	L	-	LN	W	E	L	L	GE	-

Table B10 The optimal theoretical distributions, based on the CvM metric, for modeling the total number of variants, as well as the lengths and positions of SNPs, INSs, DELs, DUPs, and DELINSs in samples of 21 cancer types from COSMIC and TCGA. Weibull = W, Gamma = G, Lognormal = LN, Cauchy = C, Logistic = L, Normal = N, Exponential = E, Geometric = GE, negative binomial = NB, Poisson = P. The symbol "-" means that all fits failed.

Database	Cancer Type	Total number of variants	SNP	INS		DEL		DUP		DELINS		
			Position	Length	Position	Length	Position	Length	Position	Length (INS)	Length (DEL)	Position
COSMIC	Breast	NB	NB	GE	NB	NB	NB	GE	NB	NB	NB	NB
	Lung	NB	NB	NB	NB	NB	NB	GE	NB	P	GE	NB
	Colon	NB	NB	GE	NB	GE	NB	GE	NB	GE	NB	NB
	Adrenal Gland	GE	NB	NB	NB	NB	NB	NB	NB	GE	NB	NB
	Biliary Tract	NB	NB	NB	NB	GE	NB	GE	NB	GE	NB	NB
	Bone	NB	NB	NB	NB	GE	NB	GE	NB	NB	NB	NB
	Cervix	NB	NB	NB	NB	NB	NB	GE	NB	GE	GE	NB
	Eye	GE	NB	NB	NB	NB	NB	GE	NB	P	GE	NB
	Kidney	NB	NB	GE	NB	GE	NB	GE	NB	NB	GE	NB
	Liver	NB	NB	GE	NB	GE	NB	GE	NB	P	NB	NB
	Oesophagus	NB	NB	NB	NB	GE	NB	GE	NB	GE	GE	NB
	Ovary	NB	NB	GE	NB	NB	NB	GE	NB	GE	GE	NB
	Pancreas	NB	NB	GE	NB	GE	NB	GE	NB	NB	GE	NB
	Pleura	NB	NB	NB	-	NB	NB	GE	NB	NB	GE	-
	Prostate	NB	NB	NB	NB	NB	NB	GE	NB	GE	NB	NB
	Skin	NB	NB	NB	NB	GE	NB	GE	NB	GE	GE	NB
	Soft Tissue	NB	NB	NB	NB	NB	NB	GE	NB	P	NB	NB
	Stomach	NB	NB	GE	NB	GE	NB	GE	NB	NB	GE	NB
	Testis	NB	NB	P	NB	NB	NB	GE	NB	GE	GE	-
	Thymus	NB	NB	NB	-	NB	NB	GE	NB	-	-	-
	Thyroid	GE	NB	NB	NB	NB	NB	GE	NB	NB	NB	-
TCGA	Breast	NB	NB	GE	NB	NB	NB	GE	NB	NB	NB	NB
	Lung	NB	NB	GE	NB	GE	NB	GE	NB	NB	NB	NB
	Colon	NB	NB	GE	NB	GE	NB	GE	NB	NB	NB	NB
	Adrenal Gland	NB	NB	GE	-	GE	NB	GE	NB	GE	NB	NB
	Biliary Tract	NB	NB	-	-	GE	NB	GE	-	GE	GE	-
	Bone	NB	NB	GE	-	NB	NB	GE	NB	GE	GE	-
	Cervix	NB	NB	GE	-	NB	NB	GE	NB	P	GE	-
	Eye	NB	NB	-	-	NB	NB	GE	-	-	-	-
	Kidney	NB	NB	NB	NB	GE	NB	GE	NB	GE	NB	NB
	Liver	NB	NB	GE	NB	NB	NB	GE	NB	NB	NB	-
	Oesophagus	NB	NB	P	-	NB	NB	GE	NB	GE	NB	-
	Ovary	NB	NB	GE	NB	NB	NB	GE	NB	NB	NB	NB
	Pancreas	NB	NB	GE	NB	NB	NB	GE	NB	NB	NB	-
	Pleura	NB	NB	NB	-	NB	NB	GE	NB	GE	GE	-
	Prostate	NB	NB	NB	-	GE	NB	GE	NB	NB	NB	-
	Skin	NB	NB	GE	NB	NB	NB	GE	NB	P	NB	NB
	Soft Tissue	GE	NB	NB	-	NB	NB	GE	NB	GE	GE	-
	Stomach	NB	NB	GE	NB	GE	NB	GE	NB	NB	NB	NB
	Testis	NB	NB	GE	-	NB	NB	GE	-	-	-	-
	Thymus	GE	NB	GE	-	GE	NB	GE	NB	-	-	-
	Thyroid	NB	NB	NB	-	GE	NB	GE	NB	GE	GE	-

Table B11 The optimal theoretical distributions, based on the CHISQ metric, for modeling the total number of variants, as well as the lengths and positions of SNPs, INSs, DELs, DUPs, and DELINSs in samples of 21 cancer types from COSMIC and TCGA. Weibull = W, Gamma = G, Lognormal = LN, Cauchy = C, Logistic = L, Normal = N, Exponential = E, Geometric = GE, negative binomial = NB, Poisson = P. The symbol "-" means that all fits failed.

Database	Cancer Type	Total number of variants	SNP	INS		DEL		DUP		DELINS		
			Position	Length	Position	Length	Position	Length	Position	Length (INS)	Length (DEL)	Position
COSMIC	Breast	LN	W	LN	NB	LN	NB/W	L	NB/W	LN	LN	NB/W
	Lung	LN	NB/W	LN	NB	W	NB	LN/L	NB/W	L	LN/GE	NB/W
	Colon	LN	W	LN	NB	LN/W	W	L	NB	LN	LN	NB
	Adrenal Gland	LN/W	NB/W	G/N	NB/L	LN	NB/W	LN	C	LN	LN/L	NB/W
	Biliary Tract	LN	W	LN	NB/W	LN/L	W	LN/L	W	LN	LN	W
	Bone	W	NB/W	LN	NB	LN	NB/W	LN/L	NB/W	LN	LN	NB/W
	Cervix	LN	NB/W	LN	NB/L	W	W	LN/L	NB/W	LN	LN/L	NB/W
	Eye	LN	NB/W	LN	NB/W	LN	NB	LN/L	W	LN	LN	NB/W
	Kidney	LN	W	LN	NB/L	LN	NB/W	LN/L	NB/W	G	LN/L	NB/W
	Liver	W	W	LN	NB	LN/L	NB/W	LN/L	NB/W	LN	W	NB
	Oesophagus	LN	LN	LN	NB/W	LN/W	NB/W	L	NB/W	LN	LN/L	LN
	Ovary	LN	W	LN	NB/W	LN	NB/W	LN/L	NB/W	L	LN/W	W
	Pancreas	LN	W	LN	NB	LN/W	NB/W	LN/L	NB/W	LN	LN	NB/W
	Pleura	LN	NB/W	LN	-	W	NB	LN/L	LN	LN/L	L	-
	Prostate	LN	W	LN	NB/W	LN/W	NB/W	LN/L	W	LN	W	NB
	Skin	W	W	LN	NB/W	LN	NB	LN/L	NB	LN	E	NB/W
	Soft Tissue	LN	W	LN	NB	LN	NB/W	LN/L	W	LN	W	NB/L
	Stomach	LN	W	LN	NB/W	LN/W	W	L	NB/W	LN/W	LN/L	LN
	Testis	LN	NB	LN/L	P	LN	LN	LN/L	LN	L	LN/L	-
	Thymus	LN	NB	LN	C	LN	W	LN/L	N/C	-	-	-
	Thyroid	LN/L	W	LN	NB	LN	NB/W	LN	NB/W	LN	W	-
TCGA	Breast	LN	NB/W	LN	NB/L	LN/W	NB/W	E	NB/W	LN	G	NB/L
	Lung	G	NB/W	LN/W	W	W	NB/W	E	NB/W	LN	LN	W
	Colon	C	W	LN/L	NB/W	LN/L	NB/W	E	NB/W	G	W	NB/C
	Adrenal Gland	LN	NB/W	LN	C	LN	NB/W	E	NB/W	LN/L	C	LN
	Biliary Tract	N	NB	E	-	LN	W/L	E	C	P/L	P/L	-
	Bone	G	NB/W	LN/L	-	LN	L	E	W	LN	E	-
	Cervix	LN	NB/W	LN	C	LN	NB/W	E	NB/W	LN	LN	C
	Eye	C	NB/W	-	-	W	LN	E	-	C	C	-
	Kidney	N	NB/W	LN	NB/L	LN/W	NB/W	E	NB/W	LN	G	LN
	Liver	LN	NB/W	LN	L	LN/W	W	E	LN/W	LN	G	-
	Oesophagus	NB/G	NB/W	LN/W	LN	LN/W	W	E	NB/W	LN	E	-
	Ovary	NB/G	NB/W	LN/L	NB/W	LN/G	NB/W	E	NB/W	LN	G/W	C
	Pancreas	L	NB	LN	NB	LN	NB/W	E	NB/W	LN	LN	-
	Pleura	L	NB/W	LN	-	LN/W	NB/W	E	C	LN/L	W/C	-
	Prostate	NB/L	NB/W	LN	C	LN/W	NB/W	E	NB/W	LN	LN	-
	Skin	W	NB/W	LN	L	LN/W	NB/W	E	NB/W	L	C	LN
	Soft Tissue	C	NB/W	LN/L	-	LN	NB/W	E	LN	C	LN	-
	Stomach	LN	W	LN/W	W	LN/W	NB/W	E	NB/W	LN	LN	L
	Testis	LN	NB	LN/L	-	LN	NB	E	-	C	C	-
	Thymus	LN	NB/W	E	-	LN/W	W	E	LN	C	C	-
	Thyroid	LN	NB/W	L	-	LN	NB	E	N/C	LN/L	P/L/GE	-

Table B12 The optimal theoretical distributions, determined by majority voting of 6 metrics (AIC, BIC, KS, AD, CvM, CHISQ), for modeling the total number of variants, as well as the lengths and positions of SNPs, INSs, DELs, DUPs, and DELINSs in samples of 21 cancer types from COSMIC and TCGA. Weibull = W, Gamma = G, Lognormal = LN, Cauchy = C, Logistic = L, Normal = N, Exponential = E, Geometric = GE, negative binomial =NB, Poisson = P. The symbol "-" means that all fits failed.

Appendix C Details about the Case Study

The toy VAR file in Fig. C2 provides examples of all variant types that can be simulated using MOV&RSIM. Additionally, it showcases all possible formats for the INS SEQ field. This VAR file contains 10 distinct variants, identified by their MIDs: "SNP1", "SNP2", "INS", "DEL", "DUP", "INV", "TRA", "DELINS", "Complex1", "Complex2". All of them are in homozygosity. Fig. 4 and Fig. C3-C8 demonstrate that MOV&RSim correctly spikes-in the variants into the template genome and, consequently, in the generated reads.

VID	MID	HAP	CHR	POS	DEL	DEL SPAN	INS	INS SEQ
V_1	SNP1	0	chr1	1’067’609	True	1	True	A
V_2	SNP1	1	chr1	1’067’609	True	1	True	A
V_3	SNP2	0	chr1	2’005’329	True	1	True	random,1
V_4	SNP2	1	chr1	2’005’329	True	1	True	random,1
V_5	INS	0	chr1	4’401’762	False	0	True	AAA
V_6	INS	1	chr1	4’401’762	False	0	True	AAA
V_7	DEL	0	chr1	4’532’177	True	5	False	None
V_8	DEL	1	chr1	4’532’177	True	5	False	None
V_9	DUP	0	chr1	5’085’337	True	2	True	hg38.fasta,chr1:5’085’338-5’085’339,3,f
V_10	DUP	1	chr1	5’085’337	True	2	True	hg38.fasta,chr1:5’085’338-5’085’339,3,f
V_11	INV	0	chr1	6’791’569	True	3	True	hg38.fasta,chr1:6’791’570-6’791’572,1,r
V_12	INV	1	chr1	6’791’569	True	3	True	hg38.fasta,chr1:6’791’570-6’791’572,1,r
V_13	TRA	0	chr1	9’436’481	True	4	False	None
V_14	TRA	1	chr1	9’436’481	True	4	False	None
V_15	TRA	0	chr1	17’566’758	False	0	True	hg38.fasta,chr1:9’436’482-9’436’485,1,f
V_16	TRA	1	chr1	17’566’758	False	0	True	hg38.fasta,chr1:9’436’482-9’436’485,1,f
V_17	DELINS	0	chr1	21’877’101	True	1	True	GGG
V_18	DELINS	1	chr1	21’877’101	True	1	True	GGG
V_19	Complex1	0	chr2	25’087’880	True	1	True	G
V_20	Complex1	1	chr2	25’087’880	True	1	True	G
V_21	Complex1	0	chr2	25’087’878	False	0	True	chr2:25’087’879-25’087’883,1,f
V_22	Complex1	1	chr2	25’087’878	False	0	True	chr2:25’087’879-25’087’883,1,f
V_23	Complex2	0	chr3	25’175’263	False	0	True	chr3:25’175’264-25’175’266,1,f
V_24	Complex2	1	chr3	25’175’263	False	0	True	chr3:25’175’264-25’175’266,1,f
V_25	Complex2	0	chr3	25’175’264	True	1	True	T
V_26	Complex2	1	chr3	25’175’264	True	1	True	T

Fig. C2 Example of MOV&RSim VAR file. SNP1 is a SNP for which the nucleotide content is chosen by the user ("A"). SNP2 is a SNP for which the nucleotide content is chosen randomly. INS is an insertion of three nucleotides ("AAA"). DEL is a deletion of 5 nucleotides. DUP is a duplication where 2 nucleotides are deleted and then re-inserted three times. INV is an inversion where three nucleotides are deleted and replaced by their reverse complement sequence. TRA is a translocation where 4 nucleotides are deleted and then re-inserted on another position. DELINS is a compound event where 1 nucleotide is deleted and 3 nucleotides ("GGG") are inserted. Complex1 is a complex event that involves both a SNP and an INS, where the INS SEQ is derived from the region previously altered by the SNP. In contrast, Complex2 is a complex event consisting of an INS followed by a SNP, where the SNP modifies the previously inserted INS SEQ.

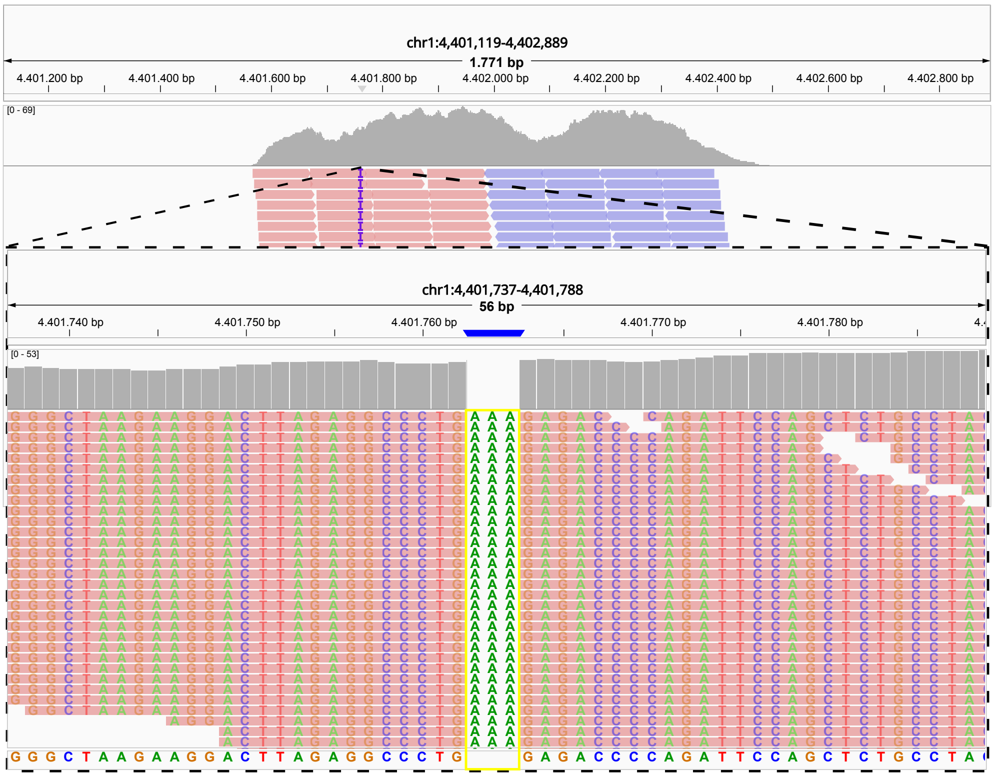

Fig. C3 IGV visualisation of simulated INS. The user defines an INS in position 4’401’763 of chromosome 1. The insertion sequence is "AAA".

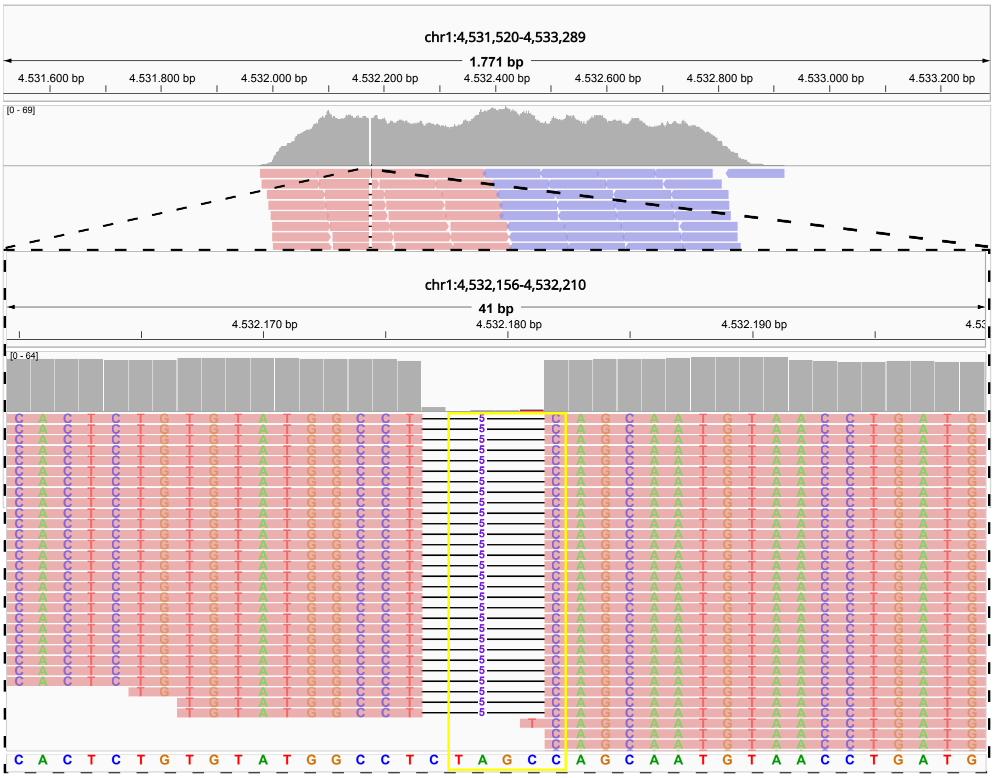

Fig. C4 IGV visualisation of simulated DEL. The user defines a DEL in position 4'532'178 of chromosome 1. The nucleotides "TAGCC" are deleted. Of note, the way reads are mapped supports a variant which is similar to the simulated variant, but not exactly the same (DEL in position 4'532'177, deleting "CTAGC").

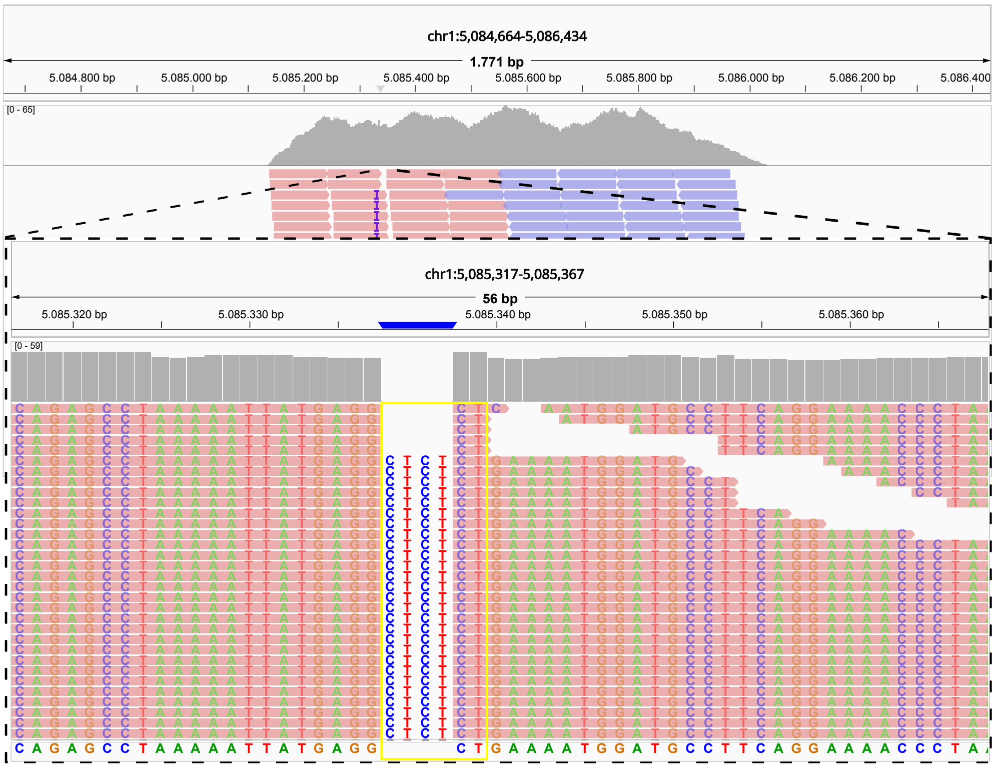

Fig. C5 IGV visualisation of simulated DUP. The user defines a DUP in position 5’085’338 of chromosome 1. The nucleotides "CT" are deleted and then re-inserted three times.

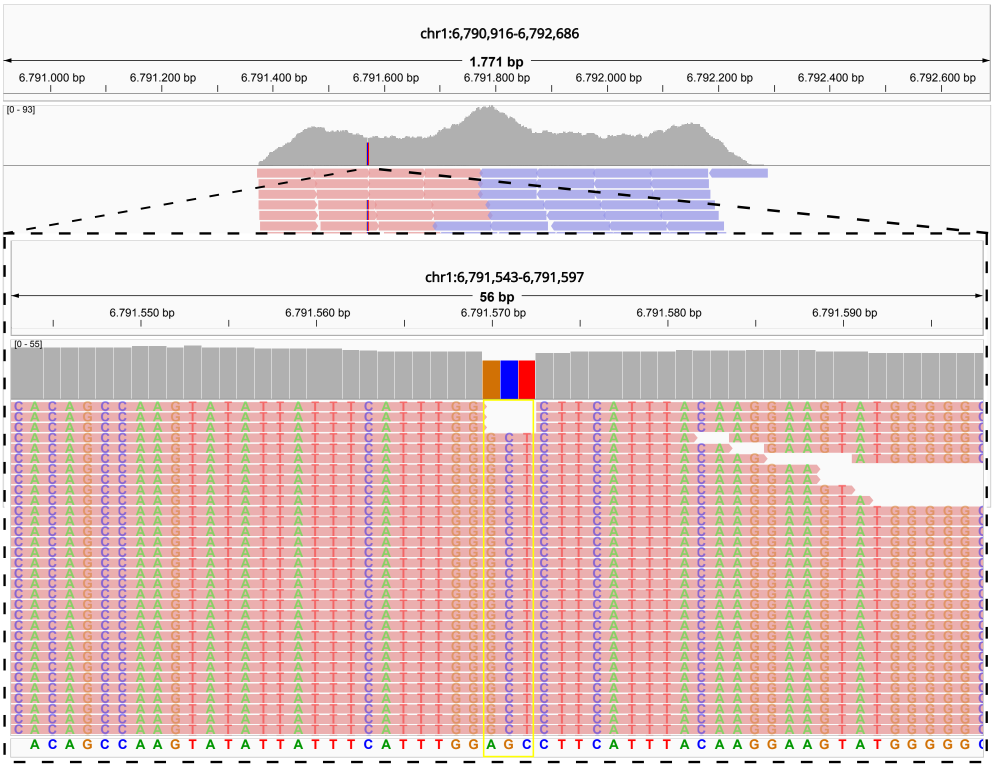

Fig. C6 IGV visualisation of simulated INV. The user defines an INV in position 6’791’570 of chromosome 1. The nucleotides "AGC" are deleted and then re-inserted in their reversed and complementary form ("GCT").

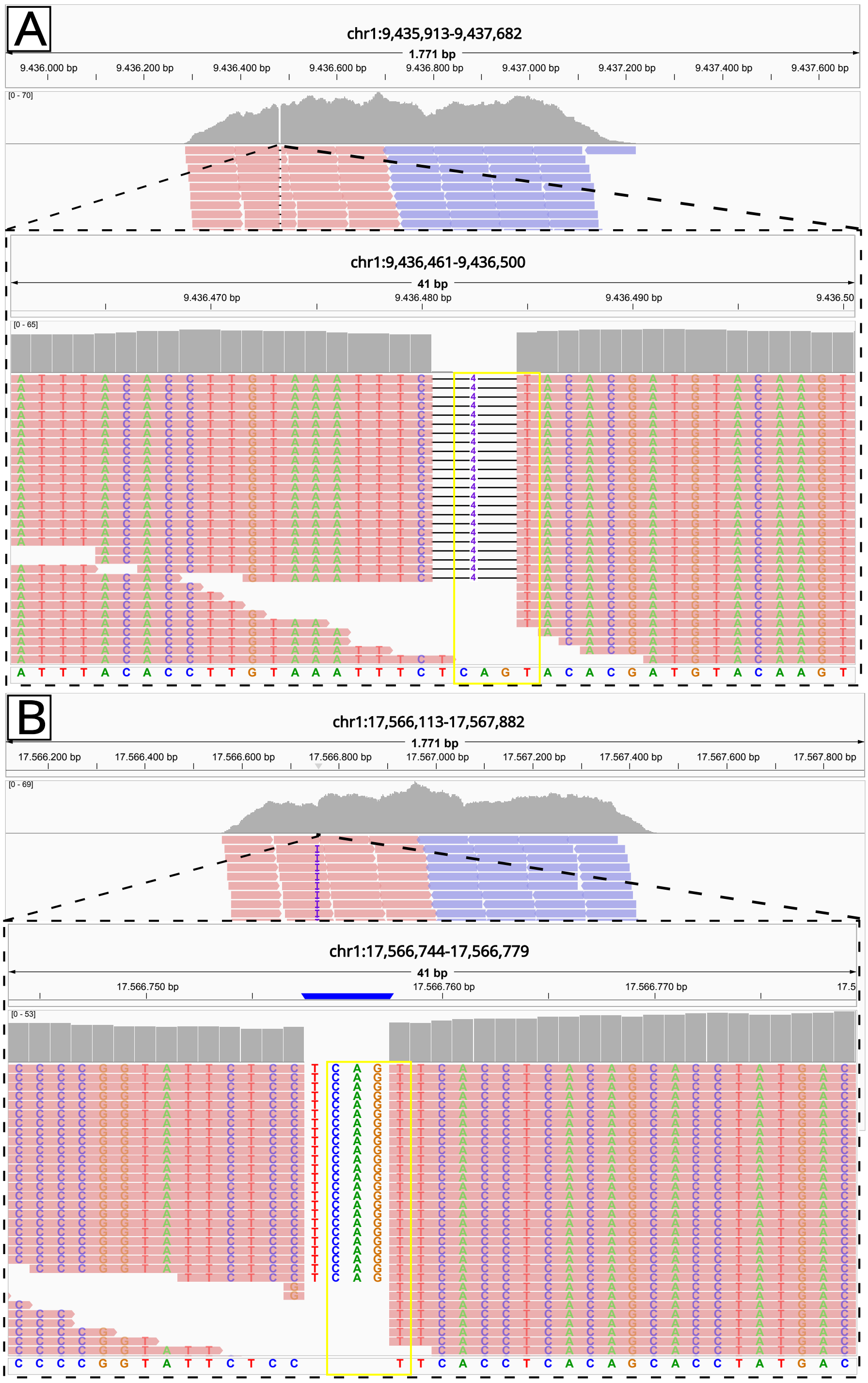

Fig. C7 IGV visualisation of simulated TRA. (A) The user defines a DEL in position 9’436’482 of chromosome 1. The nucleotides "CAGT" are deleted. (B) The user defines an INS in position 17’566’759 of chromosome 1. The insertion sequence is constituted by the same nucleotides deleted before, i.e. "CAGT". Of note, in both (A) and (B) the way reads are mapped supports a variant which is similar to the simulated variant, but not exactly the same (a DEL in position 9’436’482, deleting "TCAG" and an INS in position 17’566’758, with insertion sequence "TCAG").

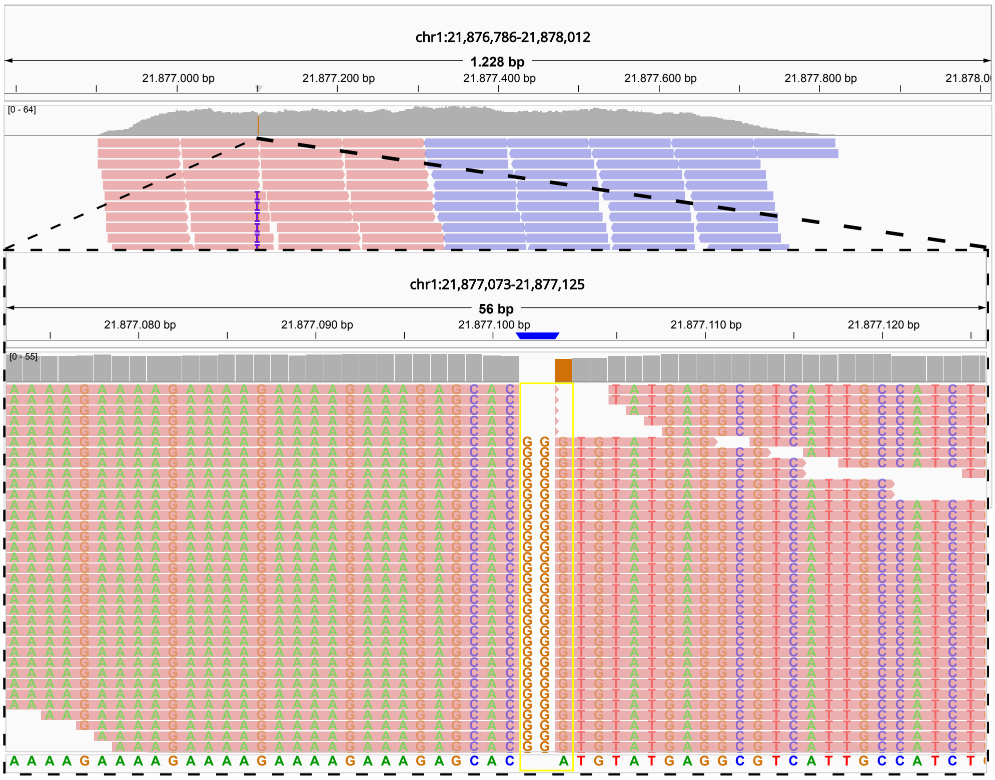

Fig. C8 IGV visualisation of simulated DELINS. The user defines a DELINS in position 21'877'102 of chromosome 1. The nucleotide "A" is deleted while the sequence "GGG" is inserted.

Appendix D Other experiments employing MOV&RSim

To show MOV&RSim's capacity to define cancer-specific variants, we executed the simulator in combination with the presets to generate 3 cancerous genomes and we analysed their mutational patterns (*first experiment*). Then, in order to show MOV&RSim's ability to simulate a wide range of reads characteristics, we selected a cancerous genome and generated its reads using information about coverage profile, sequencing errors, and base qualities learned from real NGS data. We then performed variant calling and evaluated the effect of different read characteristics on variant detection (*second experiment*). Finally, to test MOV&RSim ability to simulate clonality, we generated the genomes of four tumoral clones, mixed their reads, and performed variant calling (*third experiment*). The data and scripts used for these experiments are available at https://gitlab.com/sysbiobig/movarsim.

D.1 First Experiment: Simulation of cancer-specific mutational patterns

We used MOV&RSim's guided procedure in automatic mode to produce 3 VAR files for breast, colon, and lung cancer, respectively, using COSMIC-based presets. We set the total number of variants to 1000 and the percentage of homozygous variants to 1. Then, we run MOV&RSim genome editing block to mutate the template genome GRCh38 with the variants in the three VAR files, obtaining the corresponding altered genomes. Finally, we compared the variant characteristics in each of them. We analysed variants number per type, lengths, and positions in each of the 3 generated genomes. Proportions of variants number per type (SNP, INS, DEL, DUP, and DELINS, respectively) are reported in Fig. D9-A.

The quantity of SNPs is similar in the three samples, while the abundance of other variant types varies considerably depending on the type of tumor, according to the

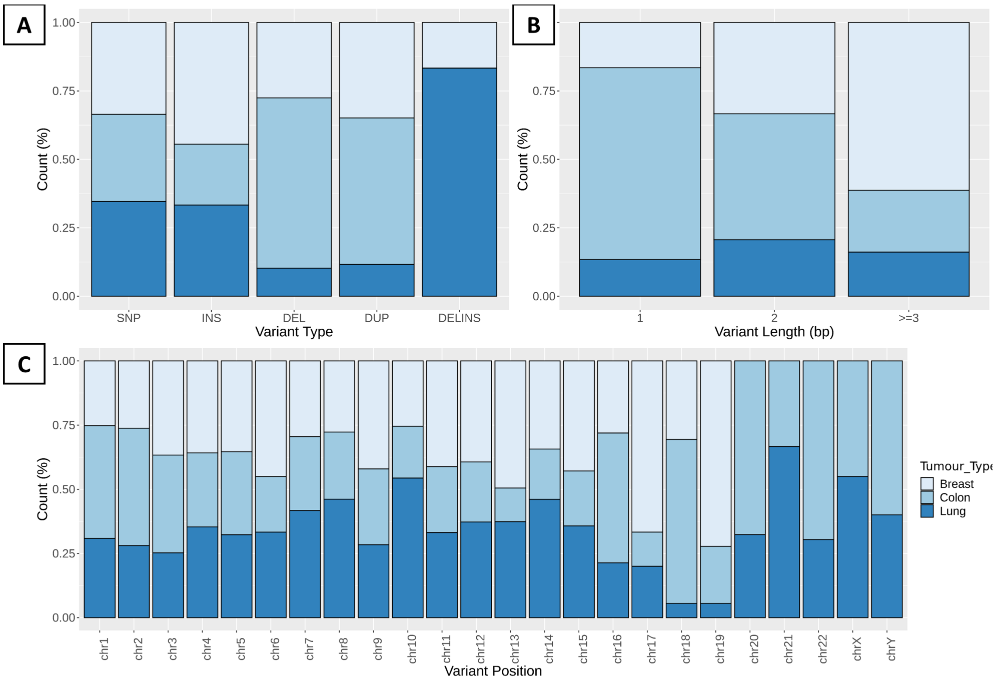

Fig. D9 Variant characteristics of 3 simulated samples, emulating breast, colon, and lung cancer samples mutational profiles. (A) shows the proportion of SNPs, INSs, DELs, DUPs, and DELINSs in each of the three tumoral samples. (B) shows proportions of variants with lengths of 1, 2, or greater or equal than 3 nucleotides in each of the three tumoral samples. (C) shows proportions of variants positions in 24 chromosomes.

results reported in Table B4. The lung cancer sample has more DELINSs compared to the breast sample and more DELINS compared to the colon sample, which has none. Figure D9-B shows the proportions of variants with lengths of 1, 2, or greater than or equal to 3 nucleotides in each of the 3 simulated samples. In this case, we observed a complete opposite characterisation of breast and colon samples. The colon sample has more variants with a length of 1 bp and fewer variants with a length of 3 or more bp compared to other cancer types. In contrast, the breast sample has many long variants ($\geq$ 3 bp length), but few variants with a length of 1 bp. Lastly, Figure D9-C illustrates the distribution of variants positions across the 24 chromosomes. It is worth noting that the generated breast cancer sample has no variants in the last 5

chromosomes. In addition, the lung cancer sample shows a lower number of variants on chromosomes 18 and 19 compared to the other two types of tumors.

In general, this analysis highlights the inherent differences among various types of cancer and the utility of our presets to easily and realistically simulate various cancer scenarios.

It is worth noting that the case study presented in this section cannot be reproduced by other simulators. No existing tool provides an automated procedure that leverages database-derived presets to populate the VAR file with variant characteristics that mirror the proportions and distributions observed in real datasets. As discussed in Appendix A, tHapMix is the only tool that partially addresses this need, as it allows a limited set of variant characteristics (specifically, the length and zygosity of DEL and DUP) to be sampled from distributions learned from a list of known variants. However, tHapMix derives these distributions from the variants of a single sample provided in a VCF file, fundamentally different from learning from databases that aggregate variants across multiple samples. The latter approach, implemented in MOV&RSim, not only provides greater statistical power to infer realistic values for variant characteristics but also enables modelling distributions specific to different sample types (e.g., cancer-type–specific datasets). In addition, MOV&RSim extends this functionality to control the number, length, position, and zygosity of SNP, INS, DEL, DUP, and DELINS variants, thereby covering a broader range of variant types and characteristics than tHapMix.

D.2 Second Experiment: Simulation of reads technical noise and its impact on variant calling performance

We used MOV&RSim to generate reads from the breast cancer genome produced within the *first experiment* (see Section D.1). To specify the regions of interest, we provided a BED file containing 400 bp regions centered on the simulated breast cancer variants (total genome of interest size $G \sim 4 \times 10^5$ bp). We choose to generate paired-end reads, with a read length $L = 100$. Also, for the final simulated sample we set the sequencing coverage to $C = 10$X.

Because all variants in this sample are homozygous (VAF = 100%), this simplified scenario represents an optimal framework to evaluate how different read characteristics affect variant calling performance, without the results being affected by the presence of low-VAF variants. For this purpose, we made MOV&RSim learn information about coverage profile (RDM), sequencing errors (PBEM), and base qualities (QM) from real data. Specifically, we gave as input three Illumina Platinum Genomes WGS BAM files [14] (HG00138, HG00139, and HG00140), the reference genome to which these samples were aligned (GRCh38), and a VCF of known SNPs from dbSNP [15]. Next, we generated three sets of reads: one using RDM, PBEM, and QM information ("control reads"), one using RDM and QM information only (reads without sequencing errors, referred to as "no-error reads"), and one using PBEM and QM information only (reads uniformly distributed across all regions and positions, referred to as "uniform-coverage reads").

Finally, we performed variant calling from the generated reads. We used the sarek pipeline [16] from nf-core [17], setting BWA-MEM2 [18] as the aligner combined with 3 different callers: HaplotypeCaller [13], FreeBayes [19], and mpileup [20]. We specifically chose germline variant callers because all variants in this experiment have a VAF of 100%. In this context, somatic callers, which are optimised to detect low-VAF variants, would have not offered any performance advantage. Moreover, we used the

default parameters and opted not to include any pre- or post-processing steps (marking duplicates, base recalibration, post-calling filtration, etc.) to evaluate the performance of the most basic pipeline.

We plotted the concordance between simulated variants' positions and called variants' positions, identifying the number of TP (simulated positions containing variants which are called as variants), FP (simulated positions not containing variants which are called as variants), and FN (simulated positions containing variants which are not called as variants). Then, using samtools [20], we calculated the coverage at all sites containing TP, FP, and FN events.

Figures D10-, D11-, D12-(A, B, C) display the concordance between the positions of simulated variants and positions of called variants, along with the number of FN, FP, and TP using the three callers on "control reads", "no-error reads", and "uniform-coverage reads", respectively. In all cases, the sum of TP and FN should equal the number of simulated variants, which is 1000. However, it actually sums to 998, indicating that 2 variants overlap with the adjacent variants.

Using "control reads" (Fig. D10), FreeBayes generates an extremely high number of FP (3696), followed by mpileup, with 277 FP. The number of FP called by HaplotypeCaller is considerably smaller (78). However, FreeBayes and mpileup call a higher number of TP (656 and 682, respectively) with respect to HaplotypeCaller (537). When using "no-error reads" (Fig. D11), the number of FP calls decreases for all the three algorithms, with a significant reduction in FreeBayes FP calls (from 3696 to 20). This outcome highlights its sensitivity to sequencing errors when executed with default parameters and without post-calling filtration, as highlighted in [21]. In contrast, the decrease in FP calls for mpileup is less pronounced. A visual inspection of the mpileup VCF revealed that many mpileup's FP calls result from its VCF formatting approach. For example, mpileup represents variants that span multiple base pairs as consecutive single-base variants in the VCF. In contrast, the MOV&RSIM ground truth VCF (as

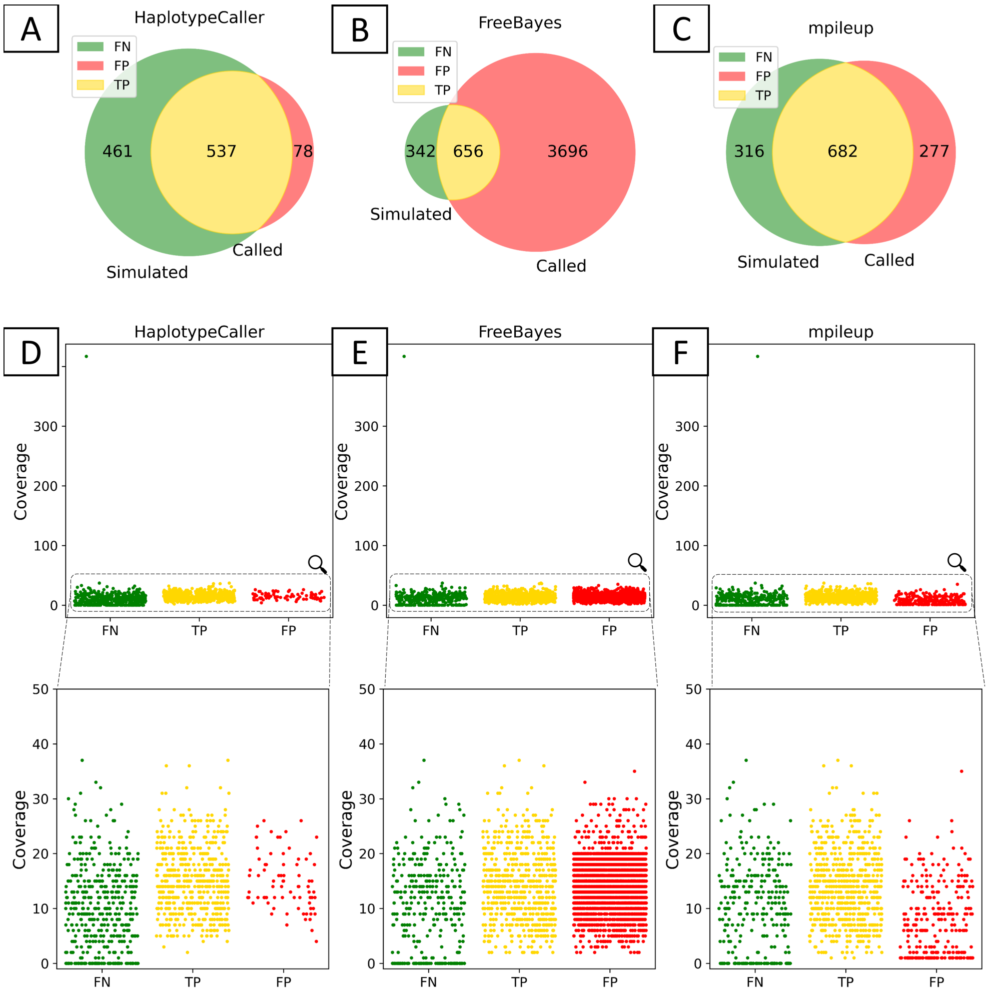

Fig. D10 Analysis of variant calling results using HaplotypeCaller, FreeBayes, and mpileup on "control reads". (A, B, C) show the concordance between the positions of simulated variants and called variants. (D, E, F) show the coverage computed at TP, FP, and FN sites.

well as the VCFs from HaplotypeCaller and Freebayes) lists only the starting position of the variants. This difference in formatting likely explains the number of FPs in mpileup, which persists even when sequencing errors are disabled. Regarding "uniform-coverage reads" (Fig. D12), FreeBayes performs better, showing fewer FN and FP, with a higher number of TP, if compared to the "control reads" scenario. In contrast, HaplotypeCaller exhibits poorer performance, with a lower TP count and higher FN

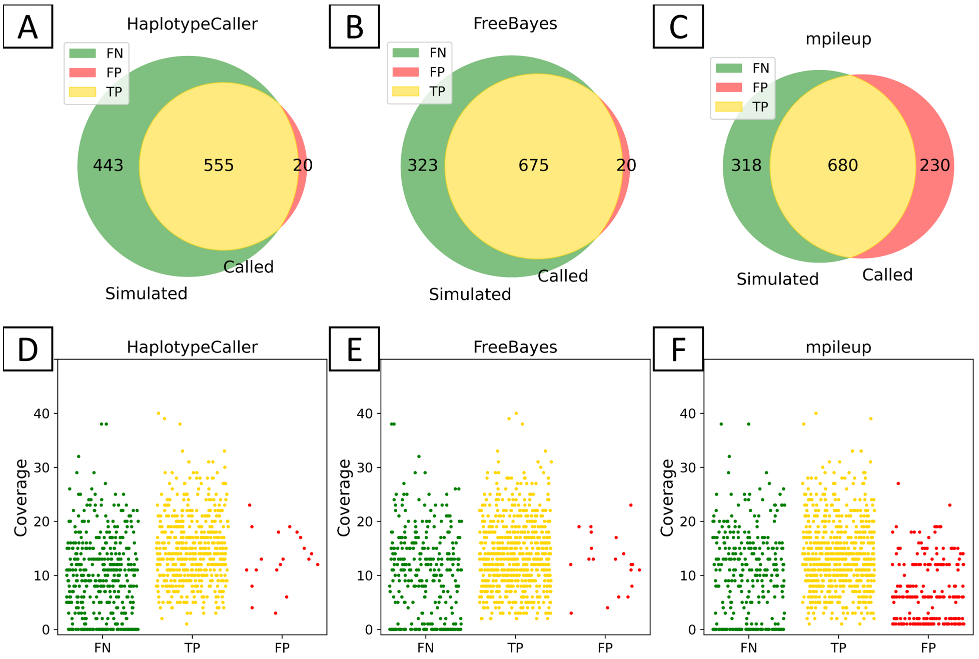

Fig. D11 Analysis of variant calling results using HaplotypeCaller, FreeBayes, and mpileup on "no-error reads". (A, B, C) show the concordance between the positions of simulated variants and called variants. (D, E, F) show the coverage computed at TP, FP, and FN sites.

and FP rates. Meanwhile, with mpileup, both TP and FP numbers increase, while FN decreases.

These performances are partially explained in Figures D10-, D11-, D12-, where it is shown the coverage of TP, FP, and FN positions using each of the three callers on each set of reads. Considering "control reads" and "no-error reads", many FN are caused by low or 0 coverage, suggesting that these variants are located in regions with low probability of observing sequencing reads in real data. With "uniform-coverage reads", FN due to low coverage are reduced. Instead, in this case, FNs that cannot be explained by low coverage become more apparent. These events are likely missed during the calling process because their nucleotide content is generated randomly. As a result, the nucleotide sequences representing these variants may, by chance, match

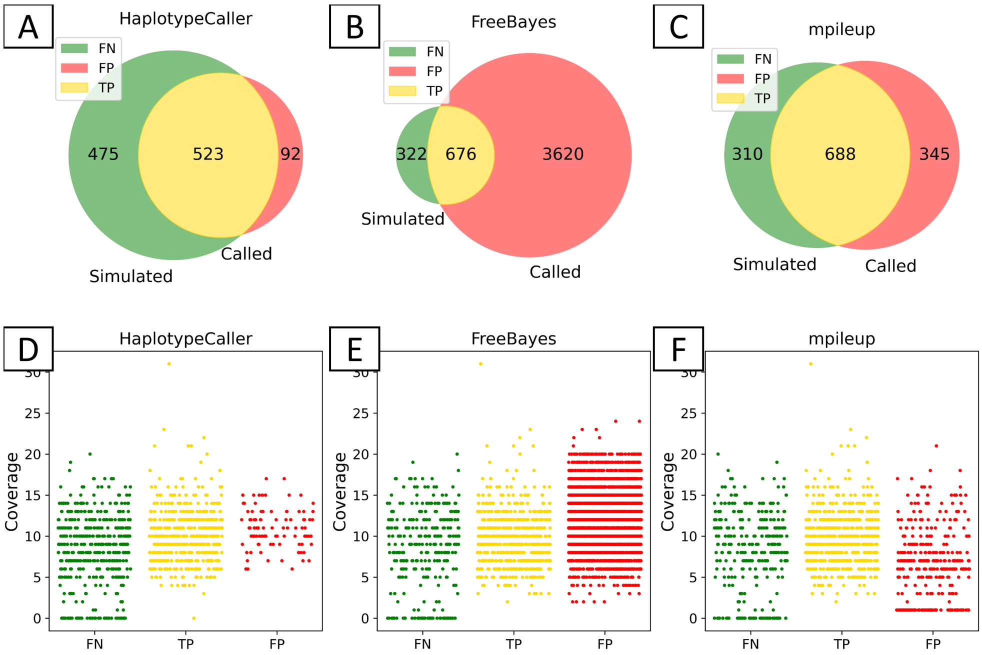

Fig. D12 Analysis of variant calling results using HaplotypeCaller, FreeBayes, and mpileup on "uniform-coverage reads". (A, B, C) show the concordance between the positions of simulated variants and called variants. (D, E, F) show the coverage computed at TP, FP, and FN sites.

the reference genome, making them undetectable by variant callers (see example in Fig. 4-B).

Remarkably, the case study presented in this section can only be reproduced using MOV&RSim. Other simulators lack essential features required for this scenario: they either do not output the mutated FASTA (Synggen, Xome-Blender, tHapMix, BAMSurgeon), do not provide the ground truth VCF (Synggen, Pysim-sv, SCNVSim, tHapMix), cannot handle overlapping variants across time (Xome-Blender, SCNVSim, VarSim, SVEngine, BAMSurgeon), or are unable to learn read characteristics such as coverage distribution, base quality scores, and sequencing error profiles from real reads (all except Synggen).

D.3 Third Experiment: Simulation of tumor clonality from input phylogenetic tree

We used MOV&RSim's guided procedure in automatic mode to generate four VAR files, each representing the variants of four clones described in an input phylogenetic tree (see Fig. D13). To generate the VAR files, we choose to rely on COSMIC presets for breast cancer and we specified as input a phylogeny found in a real breast cancer sample [22]. The "root" clone contains 1000 variants, with half of them in homozygosity and half in heterozygosity. The first clone ("clone1") has 1000 variants more than the "root", the second ("clone2") has 500 variants more than the "root", and the third ("clone3") has 500 variants more than the second. For each non-root clone, half of the additional variants are in homozygosity and half in heterozygosity.

We used the GRCh38 as template genome for variants incorporation. After generating the altered genomes for the different clones, we proceeded with read generation, following the same approach as in the previous experiment (see Section D.2). This

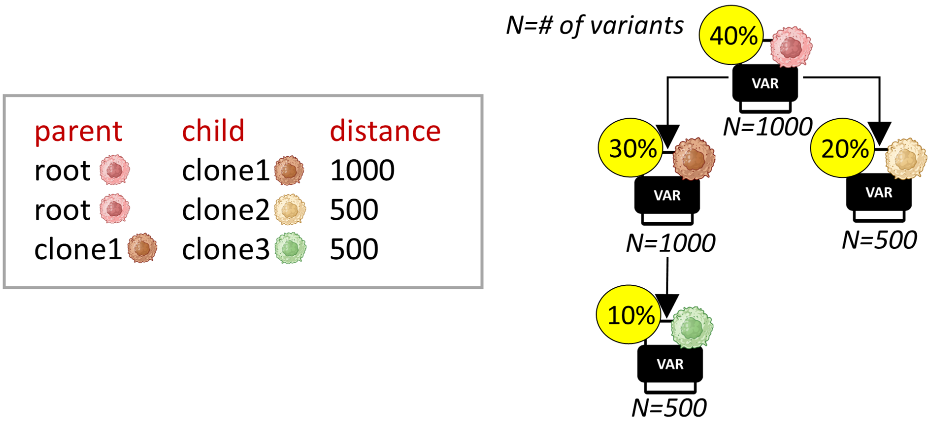

Fig. D13 Design of the phylogenetic tree for simulating a clonal breast cancer sample. The final sample is characterised by 3000 distinct variants. The variants specific to each of the four clones are evenly divided in heterozygous and homozygous. The simulated sample will include reads from each of the four clones in the following proportions: 40% from the "root", 30% from "clone1", 20% from "clone2", and 10% from "clone3".

involved learning RDM, PBEM, and QM from Illumina WGS reads (HG00138, HG00139, HG00140), specifying a BED file containing 400 bp regions centered on the simulated variants (total genome size G $\sim 2 \times 10^6$), and setting paired-end reads with a read length of L $= 100$. MOV&RSim performs these steps separately for each haplotype of the four clones and then combines the reads based on user-defined parameters (total coverage, proportions of reads generated from each haplotype, proportions of reads generated from each clone). For the final simulated sample, we set the coverage to C $= 10X$, configured the proportions of reads generated from each haplotype to 0.5 and 0.5 (no strand bias), and the proportions of reads from the four clones as 0.4, 0.3, 0.2, and 0.1, respectively. The same reads generation process is repeated three times for simulating the same sample but represented through "control reads" (using RDM, PBEM, and QM information), "no-error reads" (using RDM and QM information), and "uniform-coverage reads" (using PBEM and QM information).

For variant calling, we employed the sarek pipeline as in Section D.2 (default parameters, without pre- or post-processing steps) but used different variant callers. Given that clonal variants in this scenario are characterised by very low VAFs, we used both the germline caller HaplotypeCaller and the somatic callers Mutect2 [23] and Strelka [24]. The sum of TP and FN should be 3000 (the total number of distinct variants across the four clones), but it adds up to 2987, indicating 13 overlapping variants.

Figures D14, D15, D16 illustrate the concordance between the positions of simulated variants and called variants, as well as the coverage calculated at TP, FP, and FN sites for "control reads," "no-error reads," and "uniform-coverage reads," respectively. In all tested scenarios, Mutect2 and Strelka identify a higher number of TPs compared to HaplotypeCaller. This is outcome is expected, as the simulated clonal sample contains variants with low VAF, favoring the performance of somatic variant callers over germline variant callers.

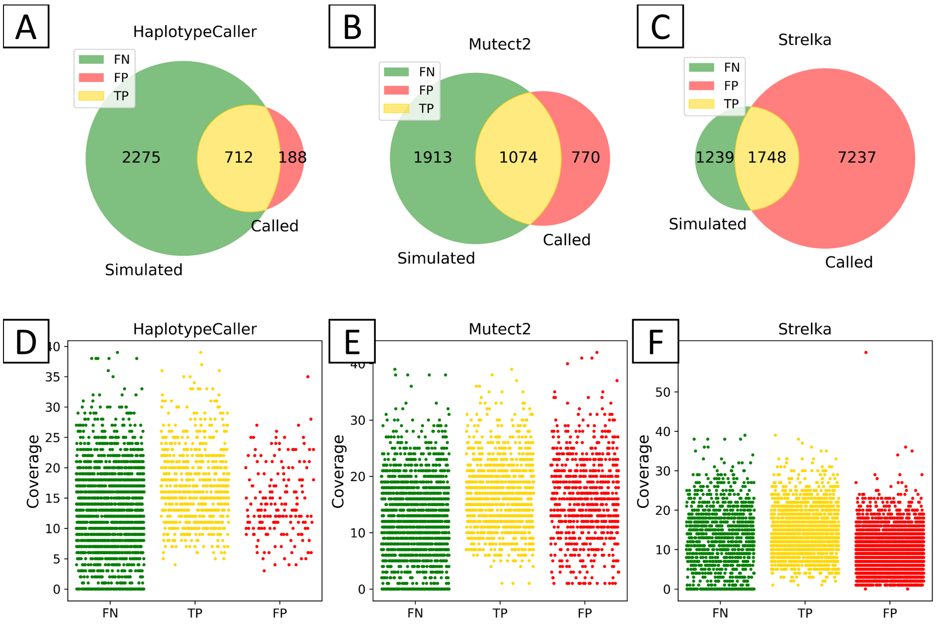

Fig. D14 Analysis of variant calling results using HaplotypeCaller, Mutect2, and Strelka on simulated “control reads”. (A, B, C) show the concordance between the positions of simulated variants and called variants. (D, E, F) show the coverage computed at TP, FP, and FN sites.

When applying variant calling to "control reads" (Fig. D14), Strelka produces a very high number of FP (7237), similar to FreeBayes in the previous experiment. Mutect2 also calls many FP (770), though fewer than TP. HaplotypeCaller, in contrast, identifies fewer FP (188). Mutect2 and Strelka report a higher number of TP (1074 and 1748, respectively) compared to HaplotypeCaller, which identifies only 712. With “no-error reads” (Fig. D15), the number of FP calls drops across all the three tools, suggesting that sequencing errors significantly impact variant calling performance. This emphasises that Mutect2 and Strelka may require fine-tuning to reduce the number of FP [21], particularly in cases like this, where tumor samples are analysed without their normal counterparts. With “uniform-coverage reads” (Fig. D16), the performance of HaplotypeCaller and Mutect2 worsen with respect to the "control reads" scenario, with a lower TP count and higher FN and FP rates. Finally, Strelka shows an increase

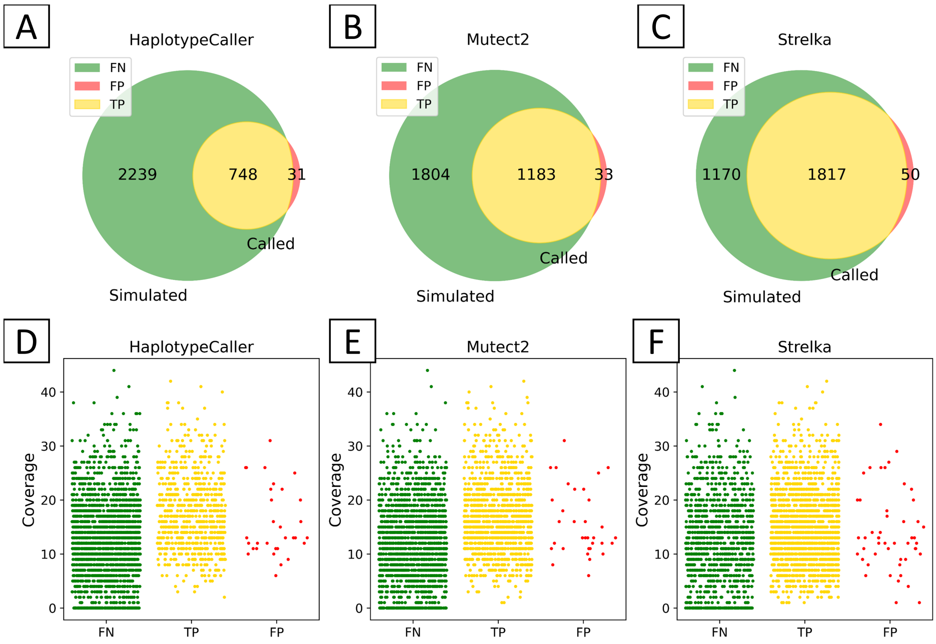

Fig. D15 Analysis of variant calling results using HaplotypeCaller, Mutect2, and Strelka on "no-error reads". (A, B, C) show the concordance between the positions of simulated variants and called variants. (D, E, F) show the coverage computed at TP, FP, and FN sites.

in TP and a decrease in FN, but this improvement comes with a higher number of FP, confirming that its algorithm is highly sensitive to sequencing errors when not fine-tuned. Consistent with previous observations, whether coverage is even or uneven has little impact, while sequencing errors remain a much more influential factor.

As in the example shown in Section D.2, MOV&RSim is the only simulator capable of producing this case study, as it uniquely combines the ability to output both the mutated FASTA and the ground truth VCF, handle overlapping variants across time, and learn read characteristics directly from real reads.

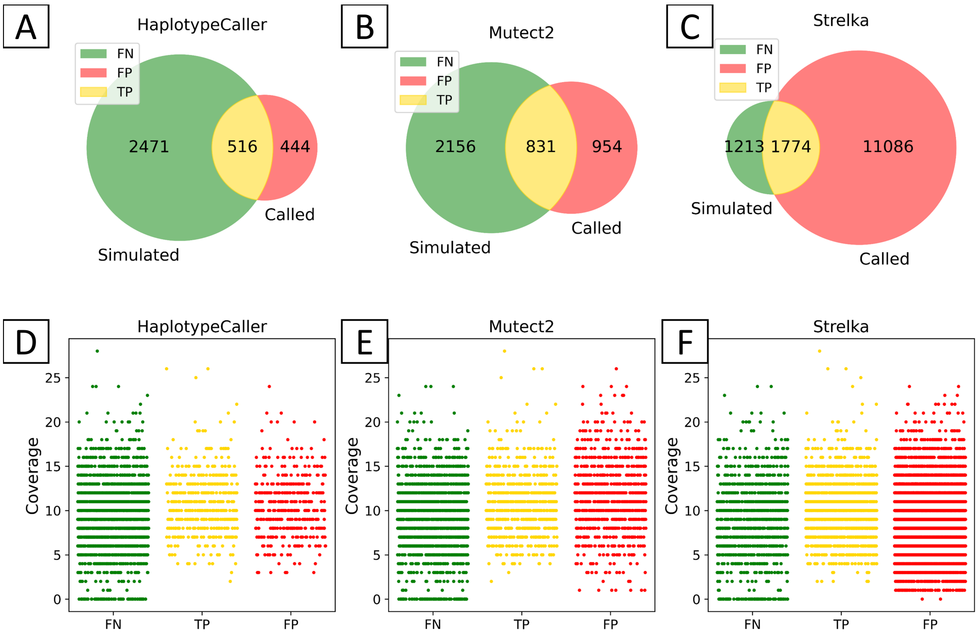

Fig. D16 Analysis of variant calling results using HaplotypeCaller, Mutect2, and Strelka on "uniform-coverage reads". (A, B, C) show the concordance between the positions of simulated variants and called variants. (D, E, F) show the coverage computed at TP, FP, and FN sites.

References

[1] Kołomański M, Szyda J, Frąszczak M, Mielczarek M. DNA sequence features underlying large-scale duplications and deletions in human. Journal of Applied Genetics. 2022;63(3):527–533.

[2] Xu C. A review of somatic single nucleotide variant calling algorithms for next-generation sequencing data. Computational and structural biotechnology journal. 2018;16:15–24.

[3] Huang W, Li L, Myers JR, Marth GT. ART: a next-generation sequencing read simulator. Bioinformatics. 2012;28(4):593–594.

[4] Kim S, Jeong K, Bafna V. Wessim: a whole-exome sequencing simulator based on in silico exome capture. Bioinformatics. 2013;29(8):1076–1077.

[5] Li H.: Wgsim. Heng Li. https://github.com/lh3/wgsim.

[6] Tanner G, Westhead DR, Droop A, Stead LF. Simulation of heterogeneous tumour genomes with HeteroGenesis and in silico whole exome sequencing. Bioinformatics. 2019;35(16):2850–2852.

[7] Xia LC, Ai D, Lee H, Andor N, Li C, Zhang NR, et al. SVEngine: an efficient and versatile simulator of genome structural variations with features of cancer clonal evolution. Gigascience. 2018;7(7):giy081.

[8] Homer N.: DWGSIM. Nils Homer. https://github.com/nh13/DWGSIM.

[9] Wang C, Yang J, Luo H, Wang K, Wang Y, Xiao ZX, et al. CancerTracer: a curated database for intrapatient tumor heterogeneity. Nucleic acids research. 2020;48(D1):D797–D806.

[10] Koboldt DC, Zhang Q, Larson DE, Shen D, McLellan MD, Lin L, et al. VarScan 2: somatic mutation and copy number alteration discovery in cancer by exome sequencing. Genome research. 2012;22(3):568–576.

[11] Ye K, Schulz MH, Long Q, Apweiler R, Ning Z. Pindel: a pattern growth approach to detect break points of large deletions and medium sized insertions from paired-end short reads. Bioinformatics. 2009;25(21):2865–2871.

[12] Ji S, Montierth MD, Wang W. MuSE: A novel approach to mutation calling with sample-specific error modeling. In: Variant Calling: Methods and Protocols. Springer; 2022. p. 21–27.

[13] McKenna A, Hanna M, Banks E, Sivachenko A, Cibulskis K, Kernytsky A, et al. The Genome Analysis Toolkit: a MapReduce framework for analyzing next-generation DNA sequencing data. Genome research. 2010;20(9):1297–1303.

[14] Zook JM, Catoe D, McDaniel J, Vang L, Spies N, Sidow A, et al. Extensive sequencing of seven human genomes to characterize benchmark reference materials. Scientific data. 2016;3(1):1–26.

[15] Sherry ST, Ward MH, Kholodov M, Baker J, Phan L, Smigielski EM, et al. dbSNP: the NCBI database of genetic variation. Nucleic acids research. 2001;29(1):308–311.

[16] Garcia M, Juhos S, Larsson M, Olason PI, Martin M, Eisfeldt J, et al. Sarek: A portable workflow for whole-genome sequencing analysis of germline and somatic variants. F1000Research. 2020;9.

[17] Ewels PA, Peltzer A, Fillinger S, Patel H, Alneberg J, Wilm A, et al. The nf-core framework for community-curated bioinformatics pipelines. Nature biotechnology. 2020;38(3):276–278.

[18] Vasimuddin M, Misra S, Li H, Aluru S. Efficient architecture-aware acceleration of BWA-MEM for multicore systems. In: 2019 IEEE international parallel and distributed processing symposium (IPDPS). IEEE; 2019. p. 314–324.

[19] Garrison E, Marth G. Haplotype-based variant detection from short-read sequencing. arXiv preprint arXiv:12073907. 2012;.

[20] Danecek P, Bonfield JK, Liddle J, Marshall J, Ohan V, Pollard MO, et al. Twelve years of SAMtools and BCFtools. Gigascience. 2021;10(2):giab008.

[21] Barbitoff YA, Abasov R, Tvorogova VE, Glotov AS, Predeus AV. Systematic benchmark of state-of-the-art variant calling pipelines identifies major factors affecting accuracy of coding sequence variant discovery. BMC genomics. 2022;23(1):155.

[22] Lomakin A, Svedlund J, Strell C, Gataric M, Shmatko A, Rukhovich G, et al. Spatial genomics maps the structure, nature and evolution of cancer clones. Nature. 2022;611(7936):594–602.

[23] Cibulskis K, Lawrence MS, Carter SL, Sivachenko A, Jaffe D, Sougnez C, et al. Sensitive detection of somatic point mutations in impure and heterogeneous cancer samples. Nature biotechnology. 2013;31(3):213–219.

[24] Kim S, Scheffler K, Halpern AL, Bekritsky MA, Noh E, Källberg M, et al. Strelka2: fast and accurate calling of germline and somatic variants. Nature methods. 2018;15(8):591–594.
